# AI-based virtual try-on technology and the activation of green psychological mechanisms: evidence from a digital consumer environment

**DOI:** 10.3389/fpsyg.2026.1750808

**Published:** 2026-03-18

**Authors:** Jingyan Bai, Jingwen Liang, Yanlei Gao, Xinqun Feng

**Affiliations:** 1 College of Fashion and Design, Donghua University, Shanghai, China; 2 School of Innovation and Creative Design, Shenzhen Polytechnic University, Shenzhen, China; 3 College of Textiles, Donghua University, Shanghai, China

**Keywords:** cognitive and affective processes, consumer psychology, environmental awareness, green psychological mechanisms, virtual try-on technology

## Abstract

With the rapid advancement of AI technologies, AI-driven virtual try-on (VTO) technology is increasingly transforming consumers’ online consumption patterns by effectively reducing return rates and resource waste through mechanisms such as accurate recommendations, thereby demonstrating substantial potential to influence consumers’ green consumption intentions. However, there is still a lack of systematic explanation of how the characteristics of VTO technology influence consumers’ green purchase intentions through psychological mechanisms. Grounded in the Stimulus–Organism–Response (SOR) model and the Theory of Planned Behavior (TPB), this study develops an integrated analytical framework to examine how the characteristics of VTO technology influence green purchase intention through consumers’ attitudes, subjective norms and perceived behavioral control. A mixed-methods approach combining task-based experiments and questionnaire surveys was employed and data from 424 participants were analyzed using PLS-SEM. The results indicate that visual realism, personalized recommendations and self-congruity primarily enhance green purchase intention by strengthening consumers’ green attitudes; social interactivity exerts an indirect effect by reinforcing subjective norms and perceived ease of use promotes green purchase intention by increasing perceived behavioral control, whereas the effect of feedback transparency on perceived control is not supported. Furthermore, the task-based experiments shows that consumers’ green preference for highly accurate VTO options is constrained by price premiums, while environmental awareness significantly strengthens the translation of green attitudes into green purchase intention. This study reveals the key psychological pathways through which AI-based VTO technology shifts from functional efficiency to green cognition, providing a theoretical foundation for digital retail platforms to promote sustainable consumption through psychologically informed technology design.

## 1 Introduction

With the rise of artificial intelligence (AI) and the development of immersive technologies such as augmented reality (AR), the digital retail environment is undergoing a profound transformation. The integration of AI and AR, particularly online VTO technology, has become one of the key sources of innovation and competitive advantage in e-commerce, fundamentally reshaping consumers’ shopping behaviors and their patterns of interaction with retailers (Chen et al., 2023). To enhance experiential richness and reduce uncertainty in online shopping, an increasing number of retailers have begun to deploy virtual shopping solutions, such as Amazon’s AR-based virtual shoe try-on feature and virtual applications offered by platforms including Taobao and Nike (Suresh and Soni, 2025). Existing research has increasingly focused on AI-based virtual technologies, but this work has primarily concentrated on their commercial applications and direct effects on consumers. Specifically, prior studies can be broadly categorized into three streams. First, research has emphasized the functional and applied aspects of the technology, examining iterations in algorithm optimization, visual realism and interactive design (An et al., 2025). Second, studies have investigated the commercial value of these technologies for consumer psychology and behavior, such as how VTO enhances user satisfaction, brand attitudes, and purchase intentions by increasing perceived hedonic and utilitarian value (Liu et al., 2025; Ho et al., 2025; Coelho and Imamović, 2025). Third, a growing body of forward-looking research has discussed the development potential of these technologies in future retail contexts, such as the metaverse (Zhang et al., 2025; Spais and Jain, 2025). However, a notable gap remains. Most existing studies adopt an instrumental rationality perspective, highlighting how virtual technologies function as marketing tools to facilitate sales conversion, while largely overlooking their potential to guide consumers toward sustainable fashion and green consumption behaviors.

The rapid expansion of online fashion retail has intensified the environmental problems associated with the fast fashion model. Consumers’ inability to physically interact with products creates substantial uncertainty regarding size, fit and material, which directly results in extremely high return rates (Hipólito et al., 2025). Frequent returns and exchanges not only generate significant logistics-related carbon footprints and packaging waste, but also increase pressure on inventory management and the disposal of discarded garments, running counter to the goals of sustainable development (George, 2024). Although prior research has examined the effects of environmental knowledge (Lin and Niu, 2018), social influence (Lazaric et al., 2020), and personal values (Varshneya et al., 2017), on green consumption, few studies have approached sustainability challenges in online shopping contexts from the perspective of technological intervention, particularly by exploring how advanced AI technologies can be leveraged to address these issues. More importantly, consumers’ initial motivation for using VTO is often driven by decision-making efficiency, such as selecting the correct size and avoiding trial-and-error, rather than by explicit environmental intentions. Existing research has not sufficiently elucidated the psychological mechanism underlying the transition from functional efficiency to green perception, nor has it clarified whether the green intentions activated through technology-enabled experiences remain stable when consumers face actual economic costs, such as price premiums.

Building on these research gaps, this study focuses on the mechanisms through which AI-based VTO technology influences consumers’ green consumption intentions, with particular attention to how it shapes consumers’ psychological cognition and judgment processes to guide lower-waste consumption decisions. As one of the most visually intensive and experience-dependent core product categories in e-commerce, apparel purchase decisions rely heavily on intuitive information to reduce uncertainty, making this context especially suitable for examining the psychological effects of VTO technology (Sarkis et al., 2025; Yrjölä et al., 2017). Beyond enhancing the shopping experience, VTO may also foster more rational and responsible consumption patterns by strengthening consumers’ perceived control over the decision-making process and information transparency (Gao and Liang, 2025).

It should be noted, the psychological effects of AI-driven technologies on green consumption do not operate uniformly across all consumer groups. Differences in technological familiarity, economic constraints and consumption contexts may significantly shape consumers’ psychological response mechanisms (Arce-Urriza et al., 2025). Accordingly, this study does not aim to generalize its findings indiscriminately to all consumer segments. Instead, Chinese university students are treated as an analytically appropriate population for examining the activation mechanisms of green psychological constructs in an AI-mediated consumption context. As digitally native consumers with high familiarity with online shopping technologies but relatively constrained purchasing power, this group provides a theoretically meaningful setting for observing how VTO technology reshapes green attitudes, subjective norms and perceived behavioral control under conditions characterized by both high technological involvement and salient economic friction (Gao and Liang, 2025). By focusing on this population, the study prioritizes explanatory depth regarding psychological formation processes over statistical generalization, thereby clarifying the applicability boundary of the proposed analytical framework. Rather than assuming a direct translation from intention to behavior, this study explicitly examines the boundary conditions under which the Theory of Planned Behavior operates in AI-mediated consumption contexts.

On this basis, to systematically capture the psychological pathways through which AI-based VTO technology influences green purchase intention, this study adopts the SOR model as an overarching analytical framework. Within this framework, VTO technological characteristics are conceptualized as external stimuli, consumers’ psychological reactions and cognitive changes constitute the organismic layer, and green purchase intention represents the behavioral response. In parallel, the TPB is incorporated to further specify the organismic layer, with a particular focus on the mediating roles of green attitudes, subjective norms and perceived behavioral control between technological stimuli and behavioral intention. In addition, the moderating effect of consumers’ environmental awareness on the relationship between attitudes and intention is examined. By integrating the SOR model and TPB, this study aims to elucidate how AI-based technology experiences function as psychological catalysts that activate consumers’ green cognitive structures and transform functional technological advantages into sustainable consumption intentions.

In summary, this study seeks to make the following marginal contributions: (1) This study responds to the debate on applying TPB in AI-driven contexts, specifically how TPB activates consumers’ psychological variables in technology-mediated consumption. By integrating the SOR model with TPB, it shows how VTO technology expands TPB’s applicability in tech-driven settings. (2) This study broadens the application of VTO, demonstrating how AI reduces decision uncertainty and reshapes judgment criteria, encouraging consumers to view precision purchasing as a low-waste, sustainable option. It also defines the boundaries of technological empowerment in green decision-making by exploring the non-significant relationship between transparency and perceived behavioral control, and the price premium’s impact on green choices, offering new insights into the attitude intention transformation. (3) This study offers managerial insights for digital retail platforms and fashion brands. It shows that VTO promotes sustainable consumption not through price premiums, but by reducing decision-making friction via technology integration and experience optimization, leading to more rational, low-waste decisions without significantly increasing consumer costs. This provides actionable strategies for balancing commercial performance with sustainability goals.

Based on the above discussion, this study addresses the following research questions:

1 Do AI-based VTO technological characteristics influence consumers’ green attitudes, subjective norms and perceived behavioral control?
2 How do these technological characteristics shape consumers’ TPB mechanisms, thereby influencing their green consumption intentions?
3 What moderating role does consumers’ environmental awareness play in the relationship between green attitudes and green consumption intentions?

## 2 Theoretical background and research hypotheses

### 2.1 Integrating TPB and SOR to explain green intention formation in AI-mediated consumption

TPB, as a classic framework in social psychology, posits that individuals’ behavioral intentions are jointly determined by three core cognitive determinants: attitude, subjective norms and perceived behavioral control (Ajzen, 1991). With the growing emphasis on sustainability issues, TPB has been widely applied to explain consumers’ green or pro-environmental behaviors. For example, Li and Shan applied an extended TPB to examine purchase intentions for green packaged organic food, revealing that health consciousness and environmental awareness influence purchase intention indirectly through attitude, subjective norms and perceived behavioral control (Li and Shan, 2025). Related research has also been conducted in areas such as green logistics choices (Mouloudj et al., 2025), green marketing (Sohaib et al., 2025), and AI-driven green entrepreneurial intention (Zhang and Jiang, 2025). Overall, these findings demonstrate that TPB exhibits strong theoretical applicability and explanatory power in examining socially responsible behavioral intentions elicited by specific contexts.

However, TPB is essentially an outcome-oriented cognitive structural model, whose core assumption is that individuals’ attitudes, norms and perceived control function as relatively stable psychological antecedents that can directly predict behavioral intention (Bondzie-Micah et al., 2022). This assumption faces clear challenges in highly technology-mediated and strongly interactive AI decision-making contexts. In AI-driven VTO settings, consumers’ green attitudes, subjective norms and perceived behavioral control are not simply derived from pre-existing value orientations, but are dynamically constructed through ongoing interactions with algorithmic systems (Sundar, 2020). In this context, TPB has limited capacity to address a critical question at the upstream end of its causal chain, namely, through which psychological mechanisms technological characteristics activate consumers’ previously latent sense of responsibility and perceived control (Kokash et al., 2025).

More importantly, AI-mediated consumption decisions do not unidirectionally enhance rationality or efficiency but are accompanied by significant psychological tension. Prior research indicates that when consumers confront AI decision-making systems, they often experience a coexisting state of trust and skepticism (Phan et al., 2025). On the one hand, algorithmic recommendations and data analytics may improve decision accuracy and reliability; on the other hand, system opacity, autonomy, and non-human attributes tend to elicit concerns regarding technological motives, privacy risks and the delegation of control (Phan et al., 2026). This psychological tendency, referred to as algorithm skepticism, suggests that technological efficiency alone is insufficient to directly translate into rational behavior. Recent studies further demonstrate that only when AI systems are perceived as authentic, emotionally engaging, and trustworthy are consumers likely to establish stable human–AI trust relationships, thereby forming positive attitudes and judgments of behavioral control (Phan and Bui, 2025). Accordingly, in AI contexts, the rational cognitive processes posited by TPB are in fact grounded in trust-based and affective evaluations rather than operating in isolation.

To systematically capture this complex process of psychological formation, this study introduces the SOR model as a process-oriented overarching framework. Unlike TPB, which focuses on cognitive structures, the SOR model emphasizes that behavior is not a direct outcome of external stimuli but rather a response generated through individuals’ internal psychological construction (Oktavia et al., 2025). In research on digital consumption and human–computer interaction, the SOR model has been widely applied to examine how technological features, interface design and interaction cues influence decision-making and behavioral responses by shaping individuals’ perceptions, emotions and cognitive evaluations (Koay and Tey, 2025). For example, Gao and Liang employed an extended SOR model to investigate the effects of key VTO features, such as visual vividness, interactive control, personalized configuration and perceived ease of use, on impulse purchase intention (Gao and Liang, 2025). In this study, SOR is treated as a source of generative logic rather than a parallel model to TPB. The TPB components of attitude, subjective norms, and perceived behavioral control are embedded in the organismic layer as internal rational cognitions, while key AI-driven VTO features are conceptualized as external stimuli. These stimuli do not directly produce green purchase intention but activate value evaluations, normative cognition, and control beliefs by shaping immersive experience, self-relevance, and social visibility, ultimately generating behavioral responses.

From this integrated perspective, SOR explains how technological experiences give rise to rational cognitive formation, whereas TPB delineates how these cognitions are further structured into behavioral motivation. Accordingly, AI-driven VTO can be understood as a sustainability catalyst at the psychological level, whose function lies not in directly determining behavioral outcomes but in reshaping consumers’ psychological pathways from technological skepticism to trust and rational decision-making.

In summary, while TPB has shown strong explanatory power in green consumption research, existing studies often treat attitudes, subjective norms and perceived behavioral control as relatively stable psychological foundations, with less focus on how these cognitive variables are activated and constructed in highly tech-mediated decision-making contexts (Li and Shan, 2025; Mouloudj et al., 2025). Particularly in AI-driven, interactive environments like VTO, consumers’ cognitive judgments are often dynamically generated through continuous human-computer interactions, rather than solely based on pre-existing value orientations. Additionally, current research typically assumes a frictionless environment for attitude intention translation, with limited exploration of whether the psychological mechanisms triggered by technology can still effectively translate into behavioral intentions under real-world constraints, such as economic friction (e.g., price premiums) (Jung et al., 2020). Therefore, this study aims to integrate the SOR and TPB frameworks to explore how VTO technology activates consumers’ planned behavior mechanisms and further examines its boundary conditions under real economic constraints.

### 2.2 Mechanisms of VTO technology features on planned behavior

Although VTO technology encompasses multidimensional functional characteristics, these technological attributes may not directly translate into consumers’ attitudes or behavioral responses (Bigne et al., 2020). During interactions with AI-driven VTO systems, consumers subjectively evaluate whether the technology is trustworthy, reduces uncertainty, and enhances controllability and social legitimacy. Only when these characteristics support rational judgment and align with personal values can their influence be activated (Gulati et al., 2024).

In this process, technological stimuli do not act directly on behavioral outcomes but instead indirectly shape the formation of green consumption intention by influencing consumers’ evaluations of behavioral value, social expectations and their own capability to perform the behavior (Ding and Lee, 2024). These psychological evaluations correspond to attitude, subjective norms and perceived behavioral control in TPB, respectively, and together constitute the core psychological pathways through which VTO technology influences green consumption decisions (Sujood and Siddiqui, 2022).

AI-driven VTO technology combines computer vision, artificial intelligence and augmented reality to digitally simulate the clothing trial process (Gao and Liang, 2025). In this study, VTO is conceptualized as an integrated digital tool whose psychological influence arises through several core technological features. Drawing from prior research on AI and online consumer behavior, these features include visual realism (Kim et al., 2024a; Hu et al., 2024), personalized recommendation (Bhuiyan, 2024; Perumallaplli, 2012), Self-congruity (Bowden et al., 2025), ease of use (Yusuf, 2025), feedback transparency (Singh and Chandra, 2026) and social interactivity (Chatterjee et al., 2025; Zhang et al., 2024). Existing research has primarily examined the effects of these technological characteristics from the perspectives of experience enhancement or purchase conversion (Arachchi and Samarasinghe, 2023). In the context of green consumption, however, these characteristics are more likely to guide consumers toward more planned consumption decisions by shaping their cognitive evaluation processes.

Based on the above analysis, this study conceptualizes VTO technological characteristics as external stimuli acting on the cognitive variables of consumers’ planned behavior and further proposes the following hypotheses:

*H1*: The characteristics of VTO technology have a positive impact on consumers’ planned behavior (attitude, subjective norm and perceived behavioral control).

#### 2.2.1 Visual realism, personalized recommendation and self-congruity in relation to consumer attitude

In online apparel shopping, although consumers are generally aware of the importance of reducing returns and waste, their rational decision-making is often constrained by uncertainty due to the lack of direct cues for assessing size and wearing outcomes (Yang and Kim, 2024). The visual realism of AI-driven VTO enhances sensory experience while serving as a digital substitute for physical try-on by providing high-fidelity cues aligned with actual product outcomes. As a key technological stimulus, it strengthens perceived information reliability and reduces concerns about trial-and-error purchasing and return-related risks (Li et al., 2025). When consumers interpret VTO results as a credible and decision-supportive information source, their purchase judgments are more likely to shift from reliance on trial-and-error to rational evaluations based on outcome predictability (Foroughi et al., 2025). In this process, consumers do not frame their purchases as environmental acts but view precision purchasing as an efficient and feasible strategy. By strengthening rational judgment and perceived controllability, visual realism aligns reduced trial-and-error purchasing and returns with green consumption goals, thereby fostering more positive green attitudes. Therefore, we propose the following hypothesis:

*H1a*: The visual realism of VTO technology has a positive effect on consumers’ attitudes toward green consumption.

Personalized recommendation can be regarded as a cognitive-level rational filtering mechanism. In traditional online shopping environments, information overload and unstructured product choices often force consumers to adopt costly trial-and-error decision processes, leading to ineffective purchases and high return rates (Wang et al., 2026). In contrast, personalized recommendations embedded in VTO systems significantly reduce the likelihood that low-fit options enter the evaluative process by filtering them out at the early stage of decision-making (Ampadu et al., 2022). Consumers’ initial acceptance of this function is primarily driven by its role in enhancing decision efficiency and reducing information burden, rather than by explicit environmental motivations (Pantano and Pizzi, 2020). However, as recommendation accuracy and usage stability improve, consumers gradually recognize that a precision matching oriented decision approach can reduce purchasing based on trial and error and lower the probability of returns (Patil, 2024). In this process, personalized recommendations do not directly activate environmental moral awareness but instead reshape standards of rational decision-making, aligning precision purchasing with low-waste green goals and fostering more positive attitudes toward technology-supported rational consumption. Therefore, we propose the following hypothesis:

*H1b*: The personalized recommendation feature of VTO technology has a positive effect on consumers’ attitudes toward green consumption.

Self-congruity reflects the degree of alignment between the VTO representation and consumers’ actual selves. Within the SOR framework, high self-congruity in VTO enhances consumers’ sense of self-involvement, thereby reshaping the psychological processing of purchase decisions (Kolańska-Stronka and Singh, 2024). Compared with traditional online shopping that relies on imagination and fosters impulse buying, highly self-congruent virtual representations shift decision focus from affective browsing to rational evaluation of long-term fit (Gao and Liang, 2025). High self-congruity strengthens consumers’ attention to the degree of correspondence between purchase outcomes and their self-image, leading precision purchasing to be perceived as a more robust decision strategy with lower reliance on trial and error, which in practice helps reduce returns caused by mismatch (Cunha, 2025). Accordingly, self-congruity promotes green consumption not by activating environmental motives directly but by reshaping evaluations of decision rationality, aligning precision purchasing with low-waste outcomes and fostering more positive green attitudes. Therefore, we propose the following hypothesis:

*H1c*: The self-congruity feature of VTO technology has a positive effect on consumers’ attitudes toward green consumption.

#### 2.2.2 Ease of use, feedback transparency and perceived behavioral control

At the implementation stage of green consumption, consumers often encounter substantial execution friction. Even when they hold intentions to reduce waste, the complexity of information processing and evaluative demands in online shopping may lead precision purchasing to be subjectively perceived as difficult to achieve, thereby weakening perceived behavioral control (Appiah Kusi et al., 2022). In AI-driven VTO contexts, perceived ease of use enhances decision feasibility by simplifying interactions and reducing cognitive load, transforming cognitively demanding judgment tasks into low-effort operational processes (Gao and Liang, 2025). Importantly, perceived ease of use does not directly alter consumers’ value orientations; rather, by lowering execution difficulty, it enables consumers to perceive precision judgment as feasible under technological support. As perceptions of feasibility increase, consumers’ confidence in their own decision-making capabilities and execution conditions correspondingly strengthens, thereby enhancing perceived behavioral control over low-waste consumption modes (Bekaroo et al., 2018). Consequently, by reducing execution friction and strengthening feasibility judgments, perceived ease of use facilitates the practical realization of precision purchasing and results in consumption outcomes characterized by low waste and sustainability. Therefore, we propose the following hypothesis:

*H1d*: The ease of use of VTO technology has a positive effect on consumers’ perceived behavioral control over green consumption.

Feedback Transparency in VTO technology refers to the clarity and interpretability of information, such as size and fit, provided by the VTO system after the try-on. In online clothing shopping, due to the difficulty in accurately understanding product information, consumers often face high information asymmetry, which weakens their perception of control over purchase outcomes (Gao and Liang, 2025). In the absence of clear judgment criteria, purchase decisions are more likely to rely on experience or guesswork, increasing the likelihood of trial-and-error purchasing and returns. In AI-driven VTO systems, higher feedback transparency, by providing clear and interpretable try-on results, helps compensate for information gaps in the judgment process, making consumers more likely to base their evaluations on relatively clear evidence rather than vague judgments (Cheng, 2026). This feedback, based on understandable information, can enhance consumers’ sense of control over their judgment and decision-making processes, thereby improving their perception of the feasibility of precision purchasing (Veltri et al., 2023).

It is important to note that transparency in AI-driven interactions is not a single-dimensional construct. Felzmann et al. distinguish between information transparency, which focuses on outcome disclosure, and process transparency, which explains the underlying logic (Felzmann et al., 2019). This study focuses on visual outcomes in VTO, representing information transparency. While clear results enhance perceived control, the lack of algorithmic explanation (process transparency) may limit trust.

In AI-driven decision contexts, this mechanism may be influenced by algorithm complexity and system opacity. Even with more information, consumers may not always develop a strong sense of control (Sweller, 2011). Nevertheless, given the concrete and context-specific feedback afforded by VTO, feedback transparency may still function as an important technological factor influencing consumers’ green perceived behavioral control. Therefore, we propose the following hypothesis:

*H1e*: The feedback transparency of VTO technology has a positive effect on consumers’ perceived behavioral control over green consumption.

#### 2.2.3 Social interactivity and subjective norms

In traditional online shopping contexts, purchase decisions typically occur in relatively private settings, making it difficult for consumers to perceive others’ social evaluations of rational consumption or return-reducing behaviors in a timely manner. By enabling the sharing of try-on outcomes and the exchange of feedback, VTO systems enhance the social visibility of decision-making behaviors, transforming originally individualized judgments into interactive actions that can be observed and evaluated by others (Hilken et al., 2020). Consumers’ initial motivation for sharing is often driven by aesthetic self-presentation or endorsement of precise decision outcomes rather than by explicit environmental concerns. However, prior research indicates that information sharing on social media can significantly activate subjective norms, thereby promoting green consumption behaviors (Xie and Madni, 2023). Zhu et al. further demonstrate that likes and positive feedback on social platforms satisfy individuals’ needs for social acceptance and internalize external evaluations into normative cognitions regarding the desirability of such behaviors (Zhu et al., 2024). Accordingly, social interactivity does not influence behavior by imposing external pressure; instead, by increasing the social visibility of decision-making, it facilitates the gradual internalization of consumption practices that reduce trial-and-error purchasing and returns as stable green subjective norms. Therefore, we propose the following hypothesis:

*H1f*: The social interactivity of VTO technology positively influences consumers’ green subjective norms.

### 2.3 Fashion green consumption and consumer planned behavior

While TPB highlights the predictive role of attitudes, subjective norms and perceived behavioral control on behavioral intentions, existing green consumption research has increasingly noted that in real decision-making contexts, intentions do not always translate consistently into actual choices. Specifically, when price premiums or additional economic costs are involved, consumers with positive green attitudes may adjust their decisions due to cost trade-offs, resulting in a disconnect between intention and behavior. However, current research often explains this disconnect from the perspectives of values or price sensitivity, typically assuming a frictionless environment for attitude intention conversion (Jung et al., 2020; Nguyễn et al., 2025). This perspective rarely considers how economic friction (e.g., price premiums) in technology-enabled contexts acts as a boundary condition, limiting consumers’ preference for green options and altering the strength of psychological variables’ influence on behavioral intentions.

According to TPB, consumers’ behavioral intentions are jointly influenced by three core psychological constructs: attitude, subjective norms and perceived behavioral control. In the context of green fashion consumption, these constructs, respectively, reflect consumers’ value cognition toward environmentally friendly behaviors, their perception of social expectations, and their assessment of their own behavioral capability. Based on this theoretical foundation, we propose the following hypotheses:

*H2*: Consumers’ planned behavior (subjective norms, attitude and perceived behavioral control) positively influences their green purchase intention.

#### 2.3.1 Consumer attitude and green purchase intention

In traditional green consumption research, consumer attitudes are seen as stable value orientations that directly predict behavioral intentions (Jung et al., 2020). However, with the integration of AI technology into the consumption decision process, existing studies have shown that in technology-enabled contexts like VTO, the foundation and role of attitudes exhibit clear contextual dependence. Specifically, in AI-driven green consumption, attitudes are shaped more by the evaluation of the technology’s rationality, effectiveness, and control, rather than abstract environmental values (Costa et al., 2025). Green attitudes manifest as positive evaluations of precision purchasing, which is perceived as a rational choice with low risk and stable outcomes due to reduced uncertainty and optimized choices (Vlačić et al., 2021). This attitude is not solely driven by environmental motives, but by the recognition of the efficiency and feasibility of this consumption mode in terms of instrumental rationality.

When consumers perceive technology-supported precision purchasing as a rational and desirable decision, their positive attitudes are more likely to translate into green consumption intentions. However, in real decision-making contexts, this translation may still be constrained by price premiums or additional economic costs. Existing research has shown that even when consumers develop positive green attitudes, their behavioral intentions do not always translate into actual purchases. This discrepancy between attitude and behavior has been observed in green consumption contexts (Zhuo et al., 2023), with economic factors like price sensitivity further weakening the translation process (Nguyễn et al., 2025). Although prior studies have noted this disconnect, they have largely explained it from the perspectives of values or price sensitivity, with limited attention to whether price constraints in technology-enabled contexts alter the strength of the effect of consumer attitudes on behavioral intention. Based on this analysis, although the transition from attitude to intention may have contextual boundaries, consumer attitudes are still considered a key psychological antecedent to forming green consumption intentions. Therefore, the following hypothesis is proposed:

*H2a*: Consumers’ positive attitudes toward green consumption behaviors.

#### 2.3.2 Subjective norms and green purchase intention

In TPB, subjective norms are typically conceptualized as perceived external social pressure experienced by individuals (Ham et al., 2015). However, recent research suggests that in social media and highly interactive digital consumption contexts, social interaction and social evaluation can significantly activate subjective norms and influence green purchase intention, indicating that norm formation is gradually shifting from passive acceptance to active perception shaped through interactive processes (Sun and Xing, 2022). In AI-mediated, socially embedded consumption settings, green subjective norms increasingly stem from observing group-endorsed decision practices. Visualized social content enhances the social appeal of sustainable behaviors, framing green consumption as a means of social validation (Testa et al., 2021). When shared VTO outcomes receive positive feedback, they signal that precision purchasing and low trial-and-error practices are socially accepted. Rather than relying on moral pressure, social feedback internalizes these rational, low-waste practices into normative cognition, strengthening the translation of perceived norms into green purchase intention (Liao, 2024). Therefore, we propose the following hypothesis:

*H2b*: Consumers’ subjective norms positively influence their green purchase intention.

#### 2.3.3 Perceived behavioral control and green purchase intention

Perceived behavioral control is traditionally understood as arising from individuals’ prior experience or intrinsic capabilities (Li et al., 2026). However, in AI-mediated decision-making contexts, the source of perceived control increasingly shifts from personal competence to the decision support provided by technological systems. Existing research indicates that AI functionalities can significantly enhance users’ perceptions of controllability over decision processes by simplifying information processing and reducing cognitive load (Bian and Che, 2025). In VTO contexts, technological characteristics such as perceived ease of use and feedback transparency reduce operational complexity and alleviate decision uncertainty, thereby transforming purchase processes that originally relied heavily on trial and error into more controllable evaluative processes (Fan et al., 2020). When consumers perceive that technology compensates for their cognitive limitations in assessing fit, their confidence in achieving precision purchasing increases. This enhanced control arises not from moral motivation but from reduced execution barriers, as VTO lowers decision risk and makes precision purchasing and waste reduction practically feasible (Gao and Liang, 2025). Accordingly, when consumers are confident that they can control purchase outcomes with technological support, they are more likely to translate this sense of control into concrete purchase commitment, thereby strengthening green consumption intention. Therefore, we propose the following hypothesis:

*H2c*: Consumers’ perceived behavioral control has a positive influence on their green purchase intention.

### 2.4 Mediating roles of TPB constructs under the SOR model

Based on the integrated SOR and TPB framework, this study posits that the multidimensional characteristics of AI-driven VTO do not directly translate into green purchase intention but instead exert their influence indirectly by activating consumers’ cognitive evaluations and psychological judgment processes. In this mechanism, attitude, subjective norms and perceived behavioral control constitute the key psychological pathways linking external technological stimuli to behavioral responses.

In AI-driven VTO contexts, green attitudes play a central mediating role between technological characteristics and green purchase intention. Although personalized recommendations, self-congruity and visual realism primarily manifest as functional attributes that enhance efficiency and experience, they transform the pursuit of high efficiency into positive evaluations of low-waste consumption modes by reshaping consumers’ judgments of decision quality, thereby facilitating the formation of green purchase intention (Bian and Che, 2025).

Personalized recommendations mediate effects through a rational filtering mechanism by reducing information-based trial-and-error costs. As consumers experience optimized selection, they increasingly view avoiding blind search as a rational pathway, fostering positive attitudes toward reducing ineffective purchases (Gao and Liang, 2025). Self-congruity operates as a mediator through a cautious evaluation mechanism. High levels of self-congruity enhance users’ sense of self-presence, shifting the decision focus from short-term attractiveness to long-term fit (Rajagopal, 2023). This heightened self-involvement fosters a deliberative mindset, leading consumers to internalize the avoidance of incorrect purchases as value-consistent. Visual realism mediates this process through evidence-based confirmation, as high-fidelity visual cues provide reliable diagnostics and reduce decision uncertainty (Kim et al., 2024b). When consumers are confident that precision purchasing can be achieved on the basis of credible evidence, they develop trust in the feasibility of low trial-and-error consumption modes, thereby establishing positive attitudes toward green behavior (Deng et al., 2024). Therefore, we propose the following hypothesis:

*H3a*: Green attitude mediates the relationship between personalized recommendation and green purchase intention.

*H3b*: Green attitude mediates the relationship between self-congruity and green purchase intention.

*H3c*: Green attitude mediates the relationship between visual realism and green purchase intention.

In AI-mediated contexts, social interactivity reshapes the formation of subjective norms by enhancing social visibility. VTO transforms consumption decisions that were originally private into observable social behaviors. As shown by Testa et al., on visually oriented social platforms, the visibility of fashion choices enables individual decisions to be transformed into social interactions through public sharing, thereby increasing the likelihood of gaining group attention and approval. When shared VTO outcomes receive positive peer feedback, such interactions constitute a mechanism of social confirmation (Testa et al., 2021). Related studies further demonstrate that social endorsement is an important driver of visible green behaviors, as it conveys a clear signal to individuals that rational decision-making and waste reduction are not merely personal choices but consumption practices endorsed by the group (Wallace and Buil, 2023). As external social cues are internalized into intrinsic behavioral motivation, subjective norms play a key mediating role between social interactivity and green purchase intention. Therefore, we propose the following hypothesis:

*H3d*: Subjective norms mediate the relationship between social interactivity and green purchase intention.

In AI-mediated retail contexts, perceived ease of use and feedback transparency enhance perceived behavioral control by increasing judgments of precision purchasing feasibility, thereby facilitating green purchase intention. In particular, ease of use lowers execution barriers by simplifying operations and reducing cognitive load, leading consumers to believe they can effectively manage decision tasks with technological support (Fan et al., 2020). Meanwhile, feedback transparency alleviates decision uncertainty by providing clear and interpretable try-on information. Prior studies have shown that the visual feedback provided by VTO serves a critical confirmation function, significantly enhancing consumers’ perceptions of controllability over both the decision-making process and its outcomes (Gao and Liang, 2025). Accordingly, perceived behavioral control functions as a psychologically mediated mechanism of technological empowerment, transforming functional support into behavioral confidence and thereby promoting green purchase intention. Therefore, we propose the following hypothesis:

*H3e*: Perceived behavioral control mediates the relationship between ease of use and green purchase intention.

*H3f*: Perceived behavioral control mediates the relationship between feedback transparency and green purchase intention.

### 2.5 Consumer environmental awareness

Within the integrated SOR–TPB pathway, consumers’ environmental awareness not only reflects their level of concern for environmental issues but also shapes how they interpret and evaluate the green outcomes embedded in technological experiences. Although positive green attitudes constitute an important prerequisite for the formation of green consumption intention, the strength with which attitudes are translated into intention varies across individuals, with environmental awareness playing a critical moderating role in this translation process (Shen and Wang, 2022).

For consumers with high environmental awareness, the recognition that precision purchasing reduces waste is highly congruent with their intrinsic value orientations. Research by Leaniz et al. demonstrates that such deep alignment between values and attitudes significantly strengthens the behavioral directionality of attitudes, making consumers more inclined to view technology as a viable pathway to achieving environmental goals and thereby reinforcing the translation of attitudes into intention (de Martínez García Leaniz et al., 2018). By contrast, consumers with low environmental awareness tend to base attitudes on functional convenience or technological novelty. As noted by Yue et al., in the absence of stable green value anchors, the attitude–intention linkage within this group exhibits pronounced contextual fragility and is easily disrupted by external factors such as price sensitivity or operational costs, resulting in a substantially weakened effect of attitudes on green intention (Yue et al., 2020). Overall, environmental awareness does not directly shape green attitudes but strengthens the extent to which technology-enabled positive attitudes translate into green consumption intentions and behavioral commitment. Therefore, we propose the following hypothesis:

*H4*: Consumer environmental awareness moderates the relationship between consumers’ green attitude and their green purchase intention.

## 3 Research method and data collection

### 3.1 Research method

**Figure 1:**
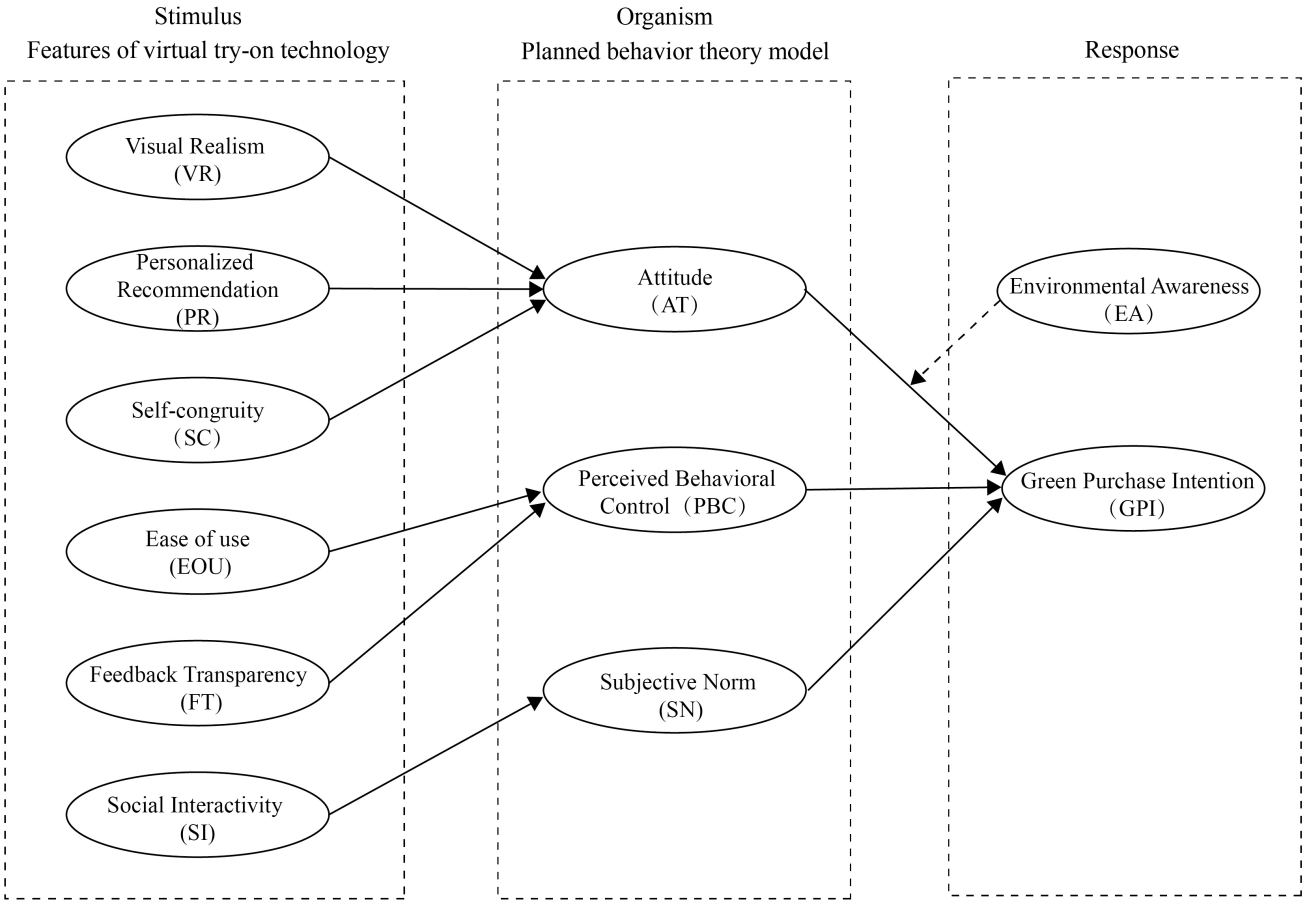
Theoretical model.

Drawing upon prior literature, TPB and the SOR model, this study constructs a new theoretical framework in which the features of AI-based VTO technology serve as the stimulus (S). Consumers’ green attitude, subjective norm and perceived behavioral control constitute the organism (O), representing the internal psychological mechanisms, while green purchase intention (GPI) functions as the response (R), reflecting the ultimate behavioral outcome. Based on this conceptualization, the proposed theoretical model is illustrated in Figure 1.

### 3.2 Participants and procedure

#### 3.2.1 Participants recruitment

Participants were recruited through the online survey platform WenJuanXin^1^, where the research purpose, participation procedures, and basic requirements were clearly explained. Eligible participants were required to meet the following criteria: (1) being enrolled university students in China and aged 18 years or older; (2) having access to functional internet-enabled devices and at least one year of online apparel shopping experience; and (3) having no prior experience with the specific VTO function examined in this study, in order to avoid familiarity-related confounds. Before the experiment began, all participants were required to read and confirm an electronic informed consent form, which outlined the study procedures, data anonymization practices and participants’ right to withdraw at any time without penalty. Only after clicking “I have read and agree” could participants proceed to the experimental interface, and this action was recorded by the system as proof of informed consent. The experimental design and data collection procedures complied with established research ethics standards. To enhance participation motivation and data quality, participants were offered an online lottery incentive upon completion of the experiment.

#### 3.2.2 Experimental procedure

This study employs a mixed research design that integrates experimental scenarios with a questionnaire survey, aiming to explore how VTO technology influences consumers’ green purchase intentions and environmental awareness through its technological features. Using the virtual fitting application on the Taobao platform as the research context, the experiment was conducted through participants’ authentic usage experiences, task-based operations and scenario-based decision tasks, thereby ensuring ecological validity and behavioral relevance.

Taobao is a leading e-commerce platform in China and one of the largest e-commerce platforms in the world (Geng et al., 2023). As one of the core product categories on the Taobao app, apparel is supported by a VTO feature that spans multiple clothing brands and categories. This function has a wide range of applications and a large user base (Gao and Liang, 2025). Its functions include personalized adjustment, intelligent size matching, dynamic try-on, outfit recommendations and social sharing. The technology is well-developed, and the user experience has been highly optimized (Lau and Ki 2021). Based on this rationale, the VTO application of the Taobao app was selected as the research context for this study.

This study selected Chinese university students as the primary participants. According to the 2025 China College Student Consumption Behavior Research Report released by iiMedia Research, China’s college student consumer market is characterized by three major trends: structural diversification, digitalization of consumption scenarios and the greening of consumption concepts. University students tend to prefer digital shopping contexts such as live-streaming commerce and show strong interest in sustainable consumption information, including green products and second-hand transactions. According to iiMedia Research, Taobao remains the most preferred e-commerce platform among university students, accounting for 39.7% of usage. This group is not only a key driver of China’s online shopping market but also among the most active adopters of emerging technologies and influential trendsetters in fashion consumption (Gao and Liang, 2025). They generally hold an open attitude toward emerging technologies such as AI and VR, exhibiting a high level of technological acceptance and fashion sensitivity. At the same time, university students typically possess a foundational awareness of green consumption concepts, showing recognition of values such as waste reduction, rational purchasing, and sustainable consumption. In summary, these characteristics make university students an appropriate and ecologically valid sample for examining how AI-based VTO technology influences the psychological mechanisms underlying green attitudes and sustainable behavioral intentions.

The experimental procedure consisted of four stages. First, during the orientation phase, participants watched a two-minute video introducing the functions and operation of Taobao’s virtual fitting room, the research purpose, data privacy protection, and the role of VTO in improving shopping accuracy and reducing returns and logistics waste. Second, in the VTO task phase, participants accessed the virtual fitting function on the Taobao mobile application using their own smartphones and completed the tasks as instructed. To ensure engagement and data validity, they were required to complete at least 10 virtual try-ons across different clothing styles and a minimum of 50 fitting operations in total, with screenshots or system records used for verification. Monitoring task completion allowed for objective assessment of participant engagement and ensured the reliability and validity of the data (Ivanov et al., 2022).

**Figure 2:**
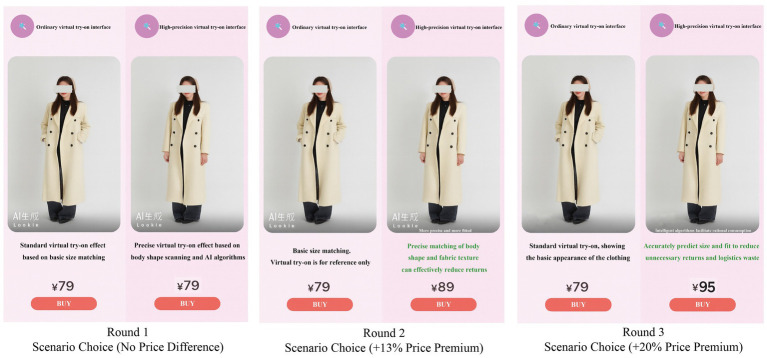
Scenario-based task selection interface.

After submitting screenshots of their virtual try-on outfits, participants completed an online questionnaire administered via WenJuanXin, a widely used survey platform in China accessed through WeChat. To examine the behavioral impact of VTO on green consumption, the questionnaire embedded scenario-based choice tasks (see Figure 2) that simulated realistic shopping contexts. Through this design, the study aimed to collect behavioral data reflecting participants’ green consumption preferences and willingness to pay a premium for sustainable products, thereby providing deeper insight into the behavioral mechanisms underlying AI-assisted green purchasing. The experiment included three choice rounds. In Round 1 (no price difference), participants chose between a standard and a high-precision VTO interface offered at the same price to assess initial preference for the environmentally friendly option. Round 2 (Approximately 13% Price Premium Test): The price for the standard interface was set at ¥79, while the high-precision interface was priced at ¥89. The scenario description emphasized that the high-precision try-on provides better body-fit accuracy and helps reduce product returns. Round 3 (Approximately 20% Price Premium Test): The price for the standard interface remained at ¥79, while the high-precision interface increased to ¥95. This round further highlighted the technology’s role in minimizing resource waste and enhancing rational purchasing behavior. Through progressively adjusted pricing scenarios, the study assessed participants’ willingness to pay for sustainability-related VTO features and examined how AI-driven precision influenced green consumption decisions under varying cost conditions. The system automatically recorded choices across all rounds and derived each participant’s maximum acceptable price premium (Willingness to Pay, WTP), which served as an indicator of behavioral intention toward green consumption. To control for order effects, both the presentation sequence of the three scenarios and the positioning of the options were randomized. Additionally, attention-check items were embedded within the task to verify participants’ attentiveness and ensure the validity and reliability of the collected data. The final stage of the study involved the questionnaire survey. A total of 451 questionnaires were collected. The first section included a screening question asking whether participants had used the virtual fitting room again after submitting screenshots; only those answering “Yes” proceeded, while others exited the survey. The second section collected demographic information, and the final section gathered data on the study variables. After removing incomplete and duplicate responses, 27 questionnaires were excluded, leaving 424 valid responses for analysis. Table 1 summarizes the participants’ demographic characteristics.

**Table 1:** Demographic statistics.

Measures	Value	Frequence	(%)
Gender	Male	188	44.34
	Female	236	55.66
Age(years)	Under 18 years old	15	3.54
	18–21	123	29.01
	22–24	153	36.08
	Above 24 years old	133	31.37
Education	Associate degree or below	123	29.01
	Bachelor’s degree	237	55.9
	Postgraduate degree or above	64	15.09
Online shopping experience	Less than 1 year	62	14.62
	1-5 years	238	56.13
	5-10 years	103	24.29
	More than 10 years	21	4.95
Monthly expenditure on online clothing purchases	Below 200 RMB	87	20.52
	201-500RMB	203	47.88
	501-1000RMB	120	28.3
	Above 1,000 RMB	14	3.3
Total		424	100

### 3.3 Scale design

The questionnaire scales used in this study were developed through an extensive literature review and adapted from established research instruments. All measurement items were assessed using a seven-point Likert scale, ranging from strongly disagree (1) to strongly agree (7). All measurement items, their original sources, and wording adaptations are reported in Table 2 and Appendix A.

**Table 2:** Measurement.

Constructs	Items	References
Visual Realism (VR)	VR 1:I find the garments presented through VTO be highly vivid.	(McLean and Wilson, 2019)
	VR 2:The texture and color of the virtual garments closely resemble those of real clothing.	(Gao and Liang, 2025)
	VR 3:The virtual model’s body features and overall proportions closely resemble my own.	
	VR 4:The virtual model’s body features and overall proportions closely resemble my own.	(Kang et al. 2020)
Personalized Recommendation (PR)	PR 1:The clothing recommended by the system aligns well with my style preferences.	(Yin et al. 2025)
	PR 2:The VTO system accurately identifies my body shape and size.	(Gao and Liang, 2025)
	PR 3:The system’s recommendations feel “just right” for me.	
	PR 4:I feel that the system understands my personalized needs.	(Yin et al. 2025)
Self-congruity(SC)	SC 1:The virtual avatar closely resembles my real-world appearance.	(Bowden et al., 2025)
	SC 2:I believe the virtual model accurately reflects my style and temperament.	(Mollel and Chen, 2025)
	SC 3:The virtual image effectively expresses my self-identity.	(Bowden et al., 2025)
	SC 4:The VTO effect is consistent with my real-life dressing experience.	
Ease of use (EOU)	EOU 1:The VTO system is very easy to operate and understand.	(Zhang and Wang, 2023)
	EOU 2:I can easily learn how to use the VTO feature.	(McLean and Wilson, 2019)
	EOU 3:The entire try-on process is efficient and effortless.	(Gao and Liang, 2025)
	EOU 4:Using the VTO technology makes me feel convenient and pleasant.	(Zhao et al. 2022)
Feedback Transparency (FT)	FT 1:The system clearly presents the basis for generating VTO results.	(Cheng, 2026)
	FT 2:I can understand the reasons behind the system’s recommendations.	(Gao and Liang, 2025)
	FT 3:The feedback provided by the system increases my trust in the try-on results.	(Pillarisetty and Mishra, 2021)
	FT 4:The system’s information presentation makes me feel that it is transparent and reliable.	(Kumar and Hajari, 2024)
Social Interactivity (SI)	SI 1:I can easily share the VTO results with my friends.	(Gao and Liang, 2025)
	SI 2:The VTO experience makes me want to interact and communicate with others.	(McLean and Wilson, 2019)
	SI 3:I enjoy showcasing my VTO experiences on social media.	(Tandon, 2023)
	SI 4:Sharing my try-on experience with others increases my desire to purchase the clothing.	(Gao and Liang, 2025)
Attitude(AT)	AT 1:I believe that rational purchasing and waste reduction are positive behaviors.	(Tandon, 2023)
	AT 2:I hold a favorable attitude toward reducing product returns through VTO.	(Angelbratt, 2025)
	AT 3:Using VTO contributes to a more sustainable way of shopping.	
	AT 4:I enjoy engaging in environmentally friendly consumption through technological means.	(Sohaib et al. 2025)
Perceived Behavioral Control(PBC)	PBC 1:I believe I am capable of making sustainable shopping decisions.	(Sharma and Paço, 2025)
	PBC 2:Using VTO makes me feel that green consumption is easier to achieve.	(Surbakti et al., 2025)
	PBC 3:I can easily determine whether the clothing fits me through VTO.	(Gao and Liang, 2025)
	PBC 4:I am confident in choosing products that are both fashionable and environmentally friendly.	(Wu and Lee, 2025)
Subjective Norm (SN)	SN 1:People around me believe that environmentally friendly consumption is commendable.	(Surbakti et al., 2025)
	SN 2:My friends and family encourage me to shop rationally and sustainably.	
	SN 3:I care about others’ opinions regarding my green consumption behavior.	(Wu and Lee, 2025)
	SN 4:When I use the VTO function, I hope others will approve of this behavior.	
Green Purchase Intention(GPI)	GPI 1:I am willing to purchase products that help reduce waste in the future.	(Surbakti et al., 2025)
	GPI 2:I plan to prioritize green and sustainable brands or products when shopping.	
	GPI 3:I actively seek information about a product’s environmental attributes or sustainable production to support my purchase decisions.	(Wu and Lee, 2025)
Environmental Awareness(EA)	EA 1:I am aware that my personal shopping behavior has a direct impact on the environment.	(Surbakti et al., 2025)
	EA 2:I believe consumers have a responsibility to reduce environmental pollution through their purchase decisions.	(Wu and Lee, 2025)
	EA 3:I pay attention to companies’ and brands’ actual practices regarding environmental protection and sustainability.	

To ensure conceptual clarity and avoid construct ambiguity, two key operational definitions warrant explicit clarification. First, green consumption in this study is operationalized as efficiency-oriented sustainability, primarily reflected in precision purchasing and return reduction enabled by AI-driven VTO technology. Rather than capturing broader value-driven or altruistic forms of green consumption (e.g., preferences for organic materials or ethical sourcing), this operationalization focuses on source reduction at the decision-making stage, which is particularly relevant in AI-mediated online apparel consumption contexts characterized by high return rates (George, 2024). Second, feedback transparency is operationalized as outcome-oriented information transparency, referring to the clarity of size and fit feedback provided after the VTO process, rather than algorithmic or process transparency (Felzmann et al., 2019).

## 4 Data analysis and results

### 4.1 Descriptive statistics

The means, standard deviations, skewness and kurtosis of all measurement items were calculated to preliminarily examine the reasonableness of the data distribution. Table 3 presents the descriptive statistical results of each item, with mean values ranging from 3.675 to 5.196. Among them, SI4 (5.196) had the highest mean, while FT2 (3.675) had the lowest. The standard deviations ranged from 1.331 to 2.266 (with GPI3 being the smallest and FT3 the largest), indicating a moderate overall degree of response dispersion. The skewness values ranged approximately from −0.987 to 0.232, and the kurtosis values from −1.567 to 0.434, all within the commonly accepted normality threshold of ±2 (Mustafy and Rahman, 2024). Therefore, the data distribution is considered reasonable and suitable for subsequent structural equation modeling (SEM) analysis.

**Table 3:** Descriptive statistics.

Constructs	Items	Mean Statistic	Std. Dev. Statistic	Skewness Statistic	Kurtosis Statistic
Visual realism (VR)	VR1	4.873	1.853	−0.657	−0.617
	VR2	4.778	1.906	−0.6	−0.747
	VR3	4.833	1.852	−0.72	−0.525
	VR4	4.821	1.91	−0.565	−0.818
Personalized recommendation (PR)	PR1	5.087	1.815	−0.852	−0.285
	PR2	5.075	1.795	−0.823	−0.346
	PR3	5.054	1.838	−0.867	−0.243
	PR4	5.04	1.886	−0.772	−0.516
Self-congruity(SC)	SC1	5.137	1.814	−0.875	−0.293
	SC2	5.059	1.84	−0.827	−0.407
	SC3	5.099	1.806	−0.93	−0.134
	SC4	5.158	1.854	−0.883	−0.277
Ease of use (EOU)	EOU1	5.118	1.843	−0.819	−0.42
	EOU2	5.052	1.875	−0.733	−0.606
	EOU3	5.191	1.806	−0.897	−0.244
	EOU4	5.052	1.858	−0.815	−0.414
Feedback transparency (FT)	FT1	3.988	2.068	0.113	−1.4
	FT2	3.675	2.255	0.232	−1.567
	FT3	3.759	2.266	0.099	−1.553
	FT4	3.715	2.166	0.21	−1.487
Social interactivity (SI)	SI1	5.165	1.793	−0.964	−0.063
	SI2	5.123	1.818	−0.9	−0.176
	SI3	5.113	1.838	−0.873	−0.303
	SI4	5.196	1.803	−0.964	−0.053
Attitude (AT)	AT1	5.04	1.829	−0.819	−0.342
	AT2	5.042	1.864	−0.892	−0.244
	AT3	5.021	1.825	−0.761	−0.448
	AT4	4.993	1.833	−0.709	−0.545
Perceived behavioral control (PBC)	PBC1	5.17	1.78	−0.931	−0.089
	PBC2	5.08	1.851	−0.855	−0.336
	PBC3	5.071	1.904	−0.896	−0.293
	PBC4	5.078	1.827	−0.847	−0.345
Subjective norm (SN)	SN1	5.118	1.869	−0.918	−0.23
	SN2	4.995	1.923	−0.761	−0.601
	SN3	5.033	1.874	−0.776	−0.51
	SN4	5.118	1.871	−0.841	−0.414
Green purchase intention (GPI)	GPI1	5.066	1.33	−0.752	0.43
	GPI2	5.068	1.34	−0.789	0.373
	GPI3	5.052	1.331	−0.688	0.387
Environmental Awareness(EA)	EA1	4.965	1.861	−0.72	−0.532
	EA2	5.106	1.78	−0.803	−0.339
	EA3	5.054	1.78	−0.841	−0.217

### 4.2 Evaluation of measurement and structural model

This study employed PLS-SEM to examine the reliability and validity of the measurement model and the results are presented in Table 4. The Cronbach’s *α* values for all constructs ranged from 0.883 to 0.940, and the composite reliability (CR) values all exceeded 0.90, indicating strong internal consistency of the measurement scales. The average variance extracted (AVE) for each latent variable was above 0.70, with the highest being 0.847 and the lowest 0.789, thus meeting the convergent validity criterion proposed by Fornell and Larcker (1981). In addition, all standardized factor loadings were greater than 0.875, suggesting strong correlations between the items and their corresponding latent constructs. Therefore, the model demonstrates good reliability and convergent validity, making it suitable for subsequent structural path analysis.

**Table 4:** Results of construct validity and reliability analysis.

Constructs	Items	Factor loading	Cronbach’s α	rho_a	Composite reliability(CR)	AVE
Visual realism (VR)	VR1	0.937	0.940	0.94	0.957	0.847
	VR2	0.927				
	VR3	0.900				
	VR4	0.916				
Personalized recommendation (PR)	PR1	0.915	0.928	0.942	0.949	0.822
	PR2	0.913				
	PR3	0.905				
	PR4	0.894				
Self-congruity(SC)	SC1	0.875	0.911	0.915	0.937	0.789
	SC2	0.891				
	SC3	0.886				
	SC4	0.901				
Ease of use (EOU)	EOU1	0.901	0.917	0.921	0.942	0.801
	EOU2	0.895				
	EOU3	0.905				
	EOU4	0.880				
Feedback transparency (FT)	FT1	0.958	0.939	1.04	0.955	0.841
	FT2	0.915				
	FT3	0.926				
	FT4	0.866				
Social interactivity (SI)	SI1	0.892	0.928	0.931	0.949	0.823
	SI2	0.913				
	SI3	0.908				
	SI4	0.915				
Attitude (AT)	AT1	0.910	0.933	0.934	0.952	0.834
	AT2	0.926				
	AT3	0.906				
	AT4	0.910				
Perceived behavioral control (PBC)	PBC1	0.914	0.925	0.928	0.946	0.815
	PBC2	0.890				
	PBC3	0.905				
	PBC4	0.902				
Subjective norm (SN)	SN1	0.912	0.933	0.935	0.952	0.833
	SN2	0.901				
	SN3	0.915				
	SN4	0.923				
Green purchase intention (GPI)	GPI1	0.907	0.903	0.906	0.939	0.838
	GPI2	0.913				
	GPI3	0.925				
Environmental awareness (EA)	EA1	0.917	0.883	0.908	0.927	0.809
	EA2	0.880				
	EA3	0.902				

This study further evaluated the validity of the measurement model from two perspectives: convergent validity and discriminant validity. First, the average variance extracted (AVE) values for all constructs ranged from 0.801 to 0.847, which are significantly higher than the recommended threshold of 0.5 and all lower than their corresponding Cronbach’s α coefficients, indicating that the measurement scales exhibit good convergent validity (Fornell and Larcker, 1981). Regarding discriminant validity, the Fornell–Larcker criterion was met, as the square roots of the AVE values for each construct (diagonal in Table 5) exceeded the corresponding interconstruct correlations, indicating satisfactory discriminant validity. In addition, all Hetero-trait–Monotrait ratio (HTMT) values were below 0.90, further supporting the model’s satisfactory discriminant validity.

**Table 5:** Discriminant validity (Fornell–Larcker criterion).

Items	AT	EA	EOU	FT	GPI	PBC	PR	SC	SI	SN	VR
AT	0.913										
EA	0.283	0.9									
EOU	0.414	0.307	0.895								
FT	0.177	0.069	0.167	0.917							
GPI	0.515	0.27	0.464	0.218	0.915						
PBC	0.308	0.269	0.278	0.119	0.416	0.903					
PR	0.272	0.302	0.356	0.101	0.413	0.333	0.907				
SC	0.346	0.285	0.383	0.174	0.439	0.291	0.322	0.888			
SI	0.36	0.265	0.304	0.173	0.422	0.308	0.262	0.337	0.907		
SN	0.292	0.31	0.349	0.155	0.422	0.326	0.341	0.35	0.337	0.913	
VR	0.31	0.231	0.23	0.052	0.406	0.243	0.229	0.251	0.226	0.269	0.92

### 4.3 Hypothesis testing

**Figure 3:**
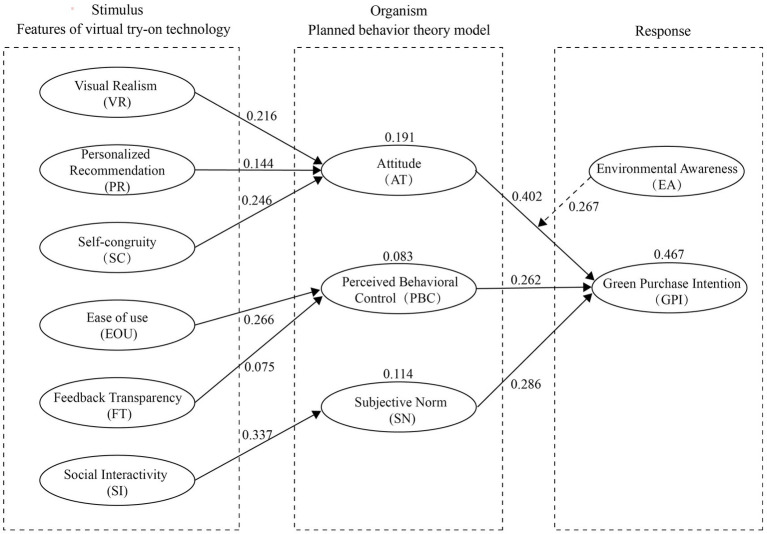
Path coefficient graph.

Before estimating path coefficients, multicollinearity among latent variables in the structural model was assessed using the Variance Inflation Factor (VIF), with values required to remain below the threshold of 5 (Dang Quan et al., 2024). The maximum VIF value in this study was 1.261, indicating that there was no issue of multicollinearity. To ensure the stability of the estimated parameters for each construct, the study employed the bootstrap resampling method, repeatedly drawing and analyzing 5,000 resampled datasets to assess the validity of the structural paths. A hypothesis was considered supported if the t-value was greater than or equal to 1.96 and the significance level (*p*-value) was less than 0.05(Zhao et al., 2022). The path coefficients are illustrated in Figure 3, while the corresponding *p*-values and t-values are presented in Table 6.

**Table 6:** Result of structural equation modeling.

Structural model paths	Original sample	Sample mean	Standard deviation	*t*-value	*p*-value	Result
H1a: VR - > AT	0.216	0.217	0.049	4.444	0	Accepted
H1b: PR - > AT	0.144	0.146	0.048	3.026	0.002	Accepted
H1c: SC - > AT	0.246	0.246	0.049	4.969	0	Accepted
H1d: EOU - > PBC	0.266	0.267	0.05	5.353	0	Accepted
H1e: FT - > PBC	0.075	0.08	0.048	1.576	0.115	Rejected
H1f: SI - > SN	0.337	0.338	0.053	6.394	0	Accepted
H2a: AT - > GPI	0.402	0.403	0.04	10.02	0	Accepted
H2b: SN - > GPI	0.286	0.284	0.042	6.838	0	Accepted
H2c: PBC - > GPI	0.262	0.263	0.036	7.285	0	Accepted

This study employed the PLS-SEM method to validate the structural model and the results are presented in Table 6. AT had a significant positive effect on GPI (*β* = 0.402, *t* = 10.020, *p* < 0.001), confirming the critical role of attitude in shaping behavioral intention as proposed in the TPB. EOU had a significant positive effect on PBC (*β* = 0.266, p < 0.001), suggesting that a convenient VTO experience enhances consumers’ perceived control over green consumption behavior. PR and VR both significantly enhanced consumer attitude (*β* = 0.144 and *β* = 0.216, respectively, *p* < 0.01), while SC also exerted a significant effect on attitude (*β* = 0.246, *p* < 0.001), highlighting the importance of system accuracy and self-identification in fostering positive green attitudes. SI had a significant influence on SN (*β* = 0.337, *p* < 0.001), and SN further had a significant positive effect on GPI (*β* = 0.286, *p* < 0.001), indicating that social influence plays a crucial role in green decision-making.

The only nonsignificant path was the effect of FT on PBC (*β* = 0.075, *p* = 0.115), indicating that information transparency alone is insufficient to enhance consumers’ sense of control. Therefore, Hypothesis H1e was not supported, while all other hypotheses were confirmed.

This study further examined the mediating effects of the core variables in TPB (AT, SN, and PBC) on the relationship between external variables and green purchase intention. The Bootstrap method (with 5,000 resamples) was employed to test five mediating paths, and the results are presented in Table 7. PBC played a significant mediating role between EOU and GPI (*β* = 0.07, *t* = 3.971, *p* < 0.001). Similarly, SN exhibited a significant mediating effect between SI and GPI (*β* = 0.096, *t* = 4.066, *p* < 0.001). Consumer green attitude also served as an important mediating variable, exerting significant mediating effects between PR and GPI (*β* = 0.058, *t* = 2.801, *p* < 0.001), between SC and GPI (*β* = 0.099, *t* = 4.126, *p* < 0.001) and between VR and GPI (*β* = 0.087, *t* = 3.929, *p* < 0.001). However, the mediating effect of Perceived Behavioral Control between FT and GPI was not significant (*β* = 0.020, *t* = 1.5, *p* = 0.134). Therefore, Hypothesis H3f was not supported, while Hypotheses H3a, H3b, H3c, H3d and H3e were supported.

**Table 7:** Mediation effects.

Structural model paths	Original sample	Sample mean	Standard deviation	*t*-value	*p*-value	Result
H3a: PR - > AT - > GPI	0.058	0.059	0.021	2.801	0.005	Accepted
H3b: SC - > AT - > GPI	0.099	0.099	0.024	4.126	0	Accepted
H3c: VR - > AT - > GPI	0.087	0.087	0.022	3.929	0	Accepted
H3d: SI - > SN - > GPI	0.096	0.097	0.024	4.066	0	Accepted
H3e: EOU - > PBC - > GPI	0.07	0.07	0.018	3.971	0	Accepted
H3f: FT - > PBC - > GPI	0.02	0.021	0.013	1.5	0.134	Rejected

This study further examined the moderating effect of EA on the relationship between AT and GPI, as shown in Table 8. The moderating path was found to be significant (*β* = 0.267, *t* = 8.218, *p* < 0.001), indicating that environmental awareness significantly strengthens the positive relationship between attitude and green purchase intention.

**Table 8:** Moderating effects of brand trust.

Structural model paths	Original sample	Sample mean	Standard deviation	*t*-value	*p*-value	Result
H4: EA × AT - > GPI	0.267	0.265	0.032	8.218	0	Accepted

### 4.4 Situational behavioral task analysis

To further examine the impact of VTO technology on consumers’ green consumption preferences at the behavioral level, this study conducted statistical and regression analyses of respondents’ choice proportions and willingness to pay, based on three rounds of scenario-based choice tasks embedded in the WenJuanXing platform.

#### 4.4.1 Analysis of choice proportions

**Figure 4:**
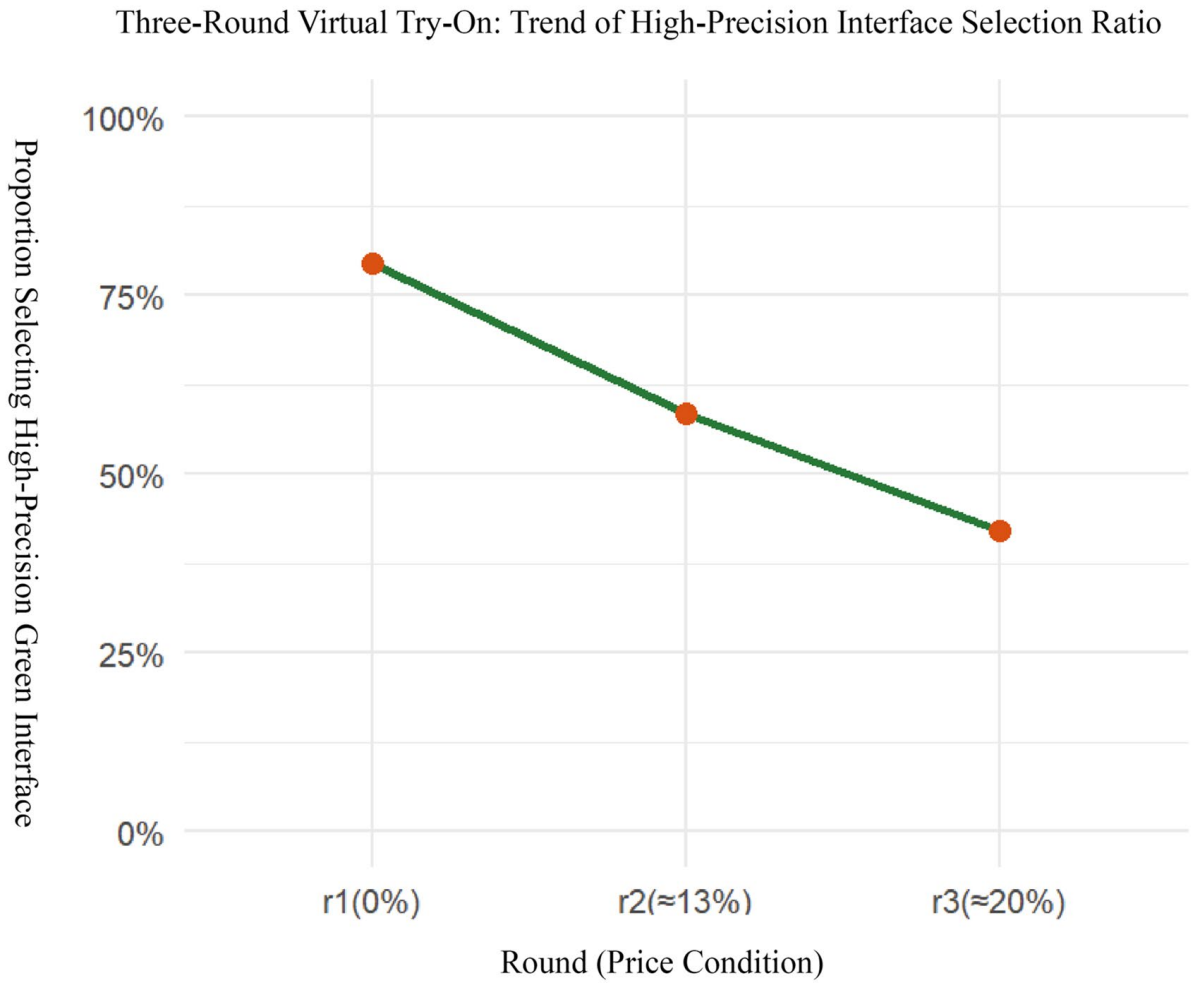
Trend of consumers’ selection ratio for high-precision virtual try-on interfaces across three rounds with increasing price differentials.

Based on the system-recorded results from the three task rounds, the proportion of each participant’s selections of the high-accuracy VTO option was calculated as the Green Preference Ratio (GPR). The results show that the overall mean GPR of the sample was approximately 0.55, indicating that most consumers tended to choose the more accurate and environmentally friendly VTO option when no price difference was involved, as illustrated in Figure 4. With the introduction of price increase scenarios (13 and 20%), the selection proportion for the green option gradually declined, revealing a trade-off between rational consumption and environmental preference. This suggests that high-accuracy VTO technology is partially perceived by consumers as a representation of green value, although its appeal remains constrained by price sensitivity.

#### 4.4.2 Results of beta regression and ordered logit regression

To more precisely characterize the mechanisms influencing the proportion of green choices, a Beta regression analysis was conducted with the GPR as the dependent variable and AT, SN, PBC and EA as independent variables. The results, presented in Table 9, indicate that none of the psychological variables exerted a statistically significant effect (*p* > 0.10). Although the coefficient for PBC was positive, suggesting that individuals with a stronger sense of control may be slightly more inclined to choose green options, the overall explanatory power of the model was weak (Pseudo *R*^2^ = 0.005). This finding is consistent with the results of the linear regression analysis, indicating that under the current experimental conditions, individuals’ proportions of green choices were primarily influenced by external factors such as price and task conditions, rather than by internal psychological attitudes or normative motivations.

**Table 9:** Beta regression results.

Predictor	Estimate	Std. error	*z*-value	*p*-value
Intercept	0.158	0.273	0.581	0.561
Attitude	0.006	0.039	0.146	0.884
Subjective norm	−0.005	0.038	0.123	0.902
Perceived behavioral control	0.034	0.025	1.365	0.172
Environmental awareness	−0.006	0.040	−0.152	0.879

Model fit indices: Pseudo *R*^2^ = 0.005, Log-likelihood = 141.3, *N* = 424.

An ordered Logit regression analysis was conducted with the Maximum Acceptable Price Premium Level (WTP_level) as the dependent variable and AT, SN, PBC, EA and their interaction term (AT × EA) as independent variables, as shown in Table 10. The results indicate that none of the four psychological variables nor their interaction term were statistically significant (*p* > 0.10). Among them, the odds ratio for PBC (OR = 1.10) was slightly greater than 1, suggesting that individuals with a stronger sense of control may be somewhat more willing to accept a higher price premium, although the effect was weak. The overall model demonstrated good fit (AIC = 768.7), and the findings were consistent with the Beta regression results, indicating that under the current experimental conditions, psychological variables had limited explanatory power for variations in consumers’ willingness-to-pay levels.

**Table 10:** Ordered logit regression results.

Predictor	Estimate	Std. error	Odds ratio (OR)	95% CI lower	95% CI upper	*p*-value
Attitude	−0.143	0.202	0.87	0.58	1.29	0.477
Subjective norm	0.023	0.069	1.02	0.89	1.17	0.743
Perceived behavioral control	0.097	0.071	1.10	0.96	1.27	0.172
Environmental awareness	−0.227	0.194	0.80	0.54	1.16	0.242
Interaction (AT × EA)	0.037	0.039	1.04	0.96	1.12	0.338

Model fit indices: AIC = 768.7, Residual deviance = 752.7, *N* = 296.

## 5 Discussion

Based on the integrated SOR–TPB framework, this study reveals how AI-driven VTO technological characteristics influence consumers’ green purchase intention through psychological mechanisms and clarifies the internal logic underlying the transformation of technological experiences into environmental motivation. The results show that VTO characteristics promote green purchase intention through three pathways: attitude, subjective norms and perceived behavioral control. Specifically, personalized recommendations, self-congruity and visual realism enhance green attitudes by strengthening self-identification and trust, whereas perceived ease of use increases perceived behavioral control by reinforcing consumers’ confidence in green decision-making. Social interactivity strengthens subjective norms, highlighting the role of social identification in green consumption. Moreover, the direct effects of certain technological characteristics indicate that the immersive and interactive nature of AI technologies can also directly stimulate green behavioral tendencies beyond cognitive mediation.

### 5.1 Limits of transparency and the formation of perceived behavioral control

Notably, feedback transparency did not significantly enhance perceived behavioral control. Prior research indicates that transparency in AI systems is multidimensional, encompassing information, process, and decision transparency (Felzmann et al., 2019). In this study, feedback transparency refers mainly to outcome-based disclosures after VTO, such as size or fit, representing a narrow form of information transparency. Prior evidence indicates that feedback lacking algorithmic explanation or actionable guidance is often perceived as unhelpful or cognitively burdensome, thereby weakening perceived control over the decision process (Sweller, 2011). Accordingly, in VTO contexts, outcome disclosure alone may be insufficient to function as effective decision support. One possible explanation is that transparency enhances cognitive understanding rather than execution capability; consumers may comprehend the information without perceiving the decision process as easier to enact. Moreover, Chinese consumers’ stronger emphasis on convenience and practicality, coupled with lower sensitivity to algorithmic transparency, may further attenuate this effect (Li et al., 2020). Overall, these findings suggest that transparent design should move beyond information visibility toward actionability to effectively strengthen perceived behavioral control.

More importantly, this finding invites a critical reconsideration of a widely held implicit assumption in AI ethics and human–AI interaction research, namely that transparency inherently enhances individuals’ perceived behavioral control. The results indicate that when transparency is confined to the visibility of outcome information, without accompanying process-level explanations or actionable decision guidance, its effects are more likely to remain at the cognitive level rather than reducing the difficulty of behavioral execution. In AI-mediated consumption contexts such as virtual try-on, perceived behavioral control is shaped less by how much information consumers receive and more by whether the technology effectively offloads cognitive effort and simplifies decision execution. Accordingly, this study refines existing transparency theories by highlighting that executability, rather than mere information visibility, constitutes the critical psychological mechanism through which AI technologies empower users and enhance perceived behavioral control.

### 5.2 Intention and behavior gap under economic friction

Using a situational behavioral task, this study shows that although SEM results confirm that technological empowerment effectively activates green purchase intention, the predictive power of psychological variables declines sharply when price trade-offs (willingness to pay, WTP) are introduced. This gap between high intention and low behavior highlights the boundary conditions of TPB in AI-mediated consumption settings characterized by salient economic friction. Theoretically, TPB primarily captures individuals’ psychological readiness, that is, attitudinal endorsement and behavioral tendencies formed under idealized conditions with insufficient consideration of external constraints (de Matos et al., 2025). VTO reduces the psychological barrier to forming green attitudes through immersive experiences, but when choices involve explicit price premiums, economic constraints substantially hinder the translation of intention into behavior.

Importantly, the pronounced price sensitivity observed in this study should not be interpreted as a mere sampling artifact associated with the use of a student population. Rather, it reflects a structurally meaningful outcome arising from the interaction between psychological activation mechanisms and specific economic boundary conditions. As digitally native consumers, university students exhibit high technological familiarity with AI-driven VTO and readily develop positive green attitudes, subjective norms and perceived behavioral control under technological support. However, their relatively constrained purchasing power renders them particularly sensitive to price premiums, thereby amplifying the disconnect between green intention and actual choice under conditions of high technological involvement combined with salient economic constraints. From this perspective, the findings suggest that in consumer segments characterized by high technological familiarity and high economic friction, TPB is more likely to manifest a pronounced intention–behavior gap. Accordingly, in AI-mediated consumption contexts, TPB is more effective in explaining the formation of green psychological readiness than in predicting behavioral realization under explicit cost constraints, thereby clarifying a key applicability boundary of intention-oriented models in technology-enabled green consumption research.

### 5.3 Value alignment and the role of TPB in AI-mediated green consumption

This study demonstrates that environmental awareness significantly strengthens the positive effect of attitudes on green intention. When technological effects resonate with individuals’ environmental value orientations, psychological efficacy is maximized. This finding corroborates value congruence theory, which posits that environmental cognition amplifies the psychological impact of technological experiences (Sapp et al., 1994).

Finally, this study emphasizes that, in AI-mediated consumption contexts, TPB is more suitable for explaining the psychological formation of green intention rather than directly predicting actual behavior. The tension between the structural model and behavioral task results provides reverse validation for this conclusion: while VTO technology effectively activates consumers’ attitudes, normative cognition and perceived behavioral control, these psychological mechanisms alone are insufficient to ensure behavioral enactment once decision-making enters a realistic choice stage involving price trade-offs. This reveals the structural boundary of intention-oriented models under conditions of high economic friction. Overall, AI technologies primarily influence sustainable consumption by lowering psychological thresholds and reshaping decision experiences, whereas their behavioral effects remain contingent on specific economic constraints and institutional conditions.

## 6 Conclusion

Based on the integrated SOR–TPB framework, this study examines how AI-driven VTO on Taobao influences green purchase intention through consumer psychological mechanisms. The results indicate that AI-driven VTO does not directly induce green behavior but operates indirectly by restructuring consumers’ rational decision-making processes. Specifically, visual realism, personalized recommendations and self-congruity strengthen green attitudes; perceived ease of use enhances perceived behavioral control; social interactivity reinforces subjective norms; whereas feedback transparency shows no significant effect. The situational choice results further indicate that consumers prefer high-precision VTO options when no price differences exist, but green choices decline as price premiums increase, suggesting that cost constraints limit green behavior. In addition, environmental awareness significantly strengthens the attitude–intention link. Overall, this study clarifies the psychological pathways through which AI-driven VTO shapes green purchase intention and highlights the practical constraints in translating green cognition into actual behavior.

### 6.1 Theoretical and managerial implications

Building on existing literature, this study advances theoretical understanding of how VTO technology shapes the psychological mechanisms of green consumption. By integrating the SOR model with the TPB, it demonstrates how VTO functions as an external stimulus that dynamically activates consumers’ green attitudes, subjective norms, and perceived behavioral control in AI-mediated consumption contexts. The findings further show that TPB constructs are not fixed prerequisites but psychological outcomes shaped during technological interaction. Importantly, this study clarifies a key boundary condition of TPB by demonstrating that under explicit economic trade-offs, the model primarily explains intention formation rather than behavioral realization. Second, regarding the theoretical debate on transparency and perceived control, the study finds that feedback transparency does not significantly enhance perceived behavioral control, challenging the implicit assumption that transparency directly equates to behavior control. This provides new theoretical evidence on the boundary conditions of technological features’ influence on control beliefs. Finally, regarding the disconnect between intention and behavior, this study employs a situational choice experiment to reveal the critical moderating role of economic costs in green decision-making, demonstrating that the translation of green attitudes into behavioral intention remains constrained by real-world factors, particularly price premiums. This finding refines the applicability of TPB by elaborating on the relationship between attitude and intention from a technological intervention perspective.

In terms of managerial practice, this study offers corrective strategic recommendations for e-commerce platforms and brands. Given consumers’ high sensitivity to price premiums, it is advised to avoid directly translating VTO’s sustainability value into immediate price markups. Instead, the focus should be on long-term value creation and risk reduction. Specifically, high-precision VTO is better suited as a core service or platform capability integrated into existing systems, generating commercial returns by enhancing overall shopping efficiency and user retention, rather than being offered as a standalone premium feature. Personalized recommendations should function as a value-guiding tool, not merely an efficiency tool, by aligning with products that have low return rates and minimal environmental impact, guiding consumers toward more sustainable choices. Moreover, the managerial significance of feedback transparency lies not in simple information disclosure, but in its ability to effectively support decision-making. Helping consumers understand the decision logic and boosting their confidence may better leverage the empowering potential of the technology.

### 6.2 Research limitations

Despite its theoretical and empirical contributions, this study has several limitations. First, this study adopts a cross-sectional design, which limits the ability to capture the dynamic evolution of consumers’ green psychological responses over repeated or long-term interactions with AI-driven virtual try-on technology. Accordingly, the findings should be interpreted as reflecting the initial psychological activation mechanisms triggered by AI-mediated consumption experiences, rather than long-term behavioral adaptation or learning effects. Second, the conceptualization of green consumption is subject to contextual boundaries. This study operationalizes green behavior primarily as return reduction and precision purchasing. Although this approach aligns with the principle of source reduction, it essentially reflects an efficiency-based form of sustainability, which differs from more proactive and altruistic green behaviors such as purchasing organic materials. Consequently, the findings primarily capture proxy environmental behavior, whose actual environmental benefits depend on the carbon costs of reverse logistics. Third, the sample composition represents an important boundary condition for interpreting the behavioral findings. The study focuses on Chinese university students, who are typically receptive to new technologies and highly engaged in digital consumption contexts such as VTO, making them suitable for examining AI-activated green psychological mechanisms. However, their relatively limited financial resources likely increase price sensitivity, potentially amplifying the price constraint effects observed in the behavioral tasks. Accordingly, the identified attitude–behavior gap may primarily reflect decision-making patterns among income-constrained or highly price-sensitive consumers. Finally, as this study is limited to apparel-related VTO applications, future research should examine other product categories to assess whether sustainable psychological mechanisms vary across consumption domains.

### 6.3 Future research directions

Future research should adopt longitudinal or experimental tracking designs to examine how sustained exposure to AI-driven VTO technology reshapes consumers’ green attitudes, perceived behavioral control, and intention–behavior consistency over time. In parallel, replicating situational behavioral tasks with samples more diverse in income and social background would help assess whether AI-activated green psychological mechanisms more reliably translate into behavior under varying economic conditions, thereby clarifying the relative roles of psychological readiness and economic constraints in the intention–behavior link. Additionally, further research is needed to refine the psychological functions of AI features by distinguishing different forms of transparency and examining their effects on perceived executability, as well as to explore the dynamic interplay between environmental awareness and technology experiences.

## Data Availability

The raw data supporting the conclusions of this article will be made available by the authors, without undue reservation.

## Footnotes

1
https://www.wjx.cn/

## References

[ref1] Ajzen I. (1991). The theory of planned behavior. Organ. Behav. Hum. Decis. Process. 50, 179–211. doi: 10.1016/0749-5978(91)90020-T

[ref2] Ampadu S. Jiang Y. Debrah E. Antwi C. O. Amankwa E. Gyamfi S. A. . (2022). Online personalized recommended product quality and E-impulse buying: a conditional mediation analysis. J. Retail. Consum. Serv. 64:102789. doi: 10.1016/j.jretconser.2021.102789

[ref3] An H. Lu W. Wu L. Peng Z. Lou J. (2025). Meta-interaction: deployable framework integrating the Metaverse and generative AI for participatory building design. Autom. Constr. 169:105893. doi: 10.1016/j.autcon.2024.105893

[ref4] Angelbratt Simon (2025) Reducing size- and fit-related returns in fashion e-commerce: exploring AI-driven virtual fitting solutions – a study on the benefits and challenges from the retailer’s perspective. Available online at: https://urn.kb.se/resolve?urn=urn:nbn:se:hb:diva-33749 (Accessed September 24, 2025).

[ref5] Appiah Kusi G. Rumki M. Z. A. Quarcoo F. H. Otchere E. Fu A. G. (2022). The role of information overload on consumers’ online shopping behavior. J. Business Manage. Stud. 4, 172–188. doi: 10.32996/jbms.2022.4.4.16

[ref6] Arachchi H. A. D. M. Samarasinghe G. D. (2023). Impulse purchase intention in an AI-mediated retail environment: extending the TAM with attitudes towards technology and innovativeness. Glob. Bus. Rev.:09721509231197721. doi: 10.1177/09721509231197721

[ref7] Arce-Urriza M. Chocarro R. Cortiñas M. Marcos-Matás G. (2025). From familiarity to acceptance: the impact of generative artificial intelligence on consumer adoption of retail Chatbots. J. Retail. Consum. Serv. 84:104234. doi: 10.1016/j.jretconser.2025.104234

[ref8] Bekaroo G. Sungkur R. Ramsamy P. Okolo A. Moedeen W. (2018). Enhancing awareness on green consumption of electronic devices: the application of augmented reality. Sustain. Energy Techn. and Assess. 30, 279–291. doi: 10.1016/j.seta.2018.10.016

[ref9] Bhuiyan M. S. (2024). The role of AI-enhanced personalization in customer experiences. J. Comput. Sci. Technol. Stud. 6, 162–169. doi: 10.32996/jcsts.2024.6.1.17

[ref10] Bian Z. Che C. (2025). How AI overview of customer reviews influences consumer perceptions in E-commerce? J. Theor. Appl. Electron. Commer. Res. 20:315. doi: 10.3390/jtaer20040315

[ref11] Bigne E. Chatzipanagiotou K. Ruiz C. (2020). Pictorial content, sequence of conflicting online reviews and consumer decision-making: the stimulus-organism-response model revisited. J. Bus. Res. 115, 403–416. doi: 10.1016/j.jbusres.2019.11.031

[ref12] Bondzie-Micah V. Qigui S. Arkorful V. E. Lugu B. K. Bentum-Micah G. Ayi-Bonte A. N. A. (2022). Predicting consumer intention to use electronic health service: an empirical structural equation modeling approach. J. Public Aff. 22:e2677. doi: 10.1002/pa.2677

[ref13] Bowden J. L.-H. Eshaghi M. S. Afshardoost M. Rahman S. M. (2025). Metaverse customer experience: conditional mediation of avatar self-congruity, user similarity and well-becoming. J. Consum. Mark. 42, 940–962. doi: 10.1108/JCM-10-2024-7298

[ref14] Chatterjee R. George S. R. Verma J. S. Heggde G. Gadhavi D. D. (2025). Impact of human and AI-agent services on customer learning, immersion and loyalty: the role of interactivity. J. Serv. Theory Pract. 35, 668–692. doi: 10.1108/JSTP-08-2024-0258

[ref15] Chen J. V. Ha Q.-A. Vu M. T. (2023). The influences of virtual reality shopping characteristics on consumers’ impulse buying behavior. Int. J. Hum.-Comput. Interact. 39, 3473–3491. doi: 10.1080/10447318.2022.2098566

[ref16] Cheng N. (2026). The impact of AR-enabled try-on experiences on consumer purchase decisions: the moderating role of AI-powered recommendation agents. Future Technol. 5, 355–365. doi: 10.55670/fpll.futech.5.1.30

[ref17] Coelho M. C. C. D. Imamović I. (2025). “AI-driven personalization in beauty retail: exploring how AI-based applications influence customer satisfaction and brand loyalty,” in Leveraging AI for Effective Digital Relationship Marketing, (IGI Global Scientific Publishing).

[ref18] Costa A. Marozzo V. Abbate T. (2025). Consumers’ attitudes toward virtual try-on technology: an extended TAM model. Int. J. Retail Distrib. Manag. 53, 184–199. doi: 10.1108/IJRDM-01-2025-0060

[ref19] Cunha M. N. (2025). Transforming online retail: the impact of augmented and virtual reality on consumer engagement and experience in E-commerce in the context of the sustainable development goals (SDG). J. Lifestyle SDGs Rev. 5:e4816–e4816. doi: 10.47172/2965-730X.SDGsReview.v5.n03.pe04816

[ref20] Dang Quan T. Tan G. W.-H. Aw E. C.-X. Cham T.-H. Basu S. Ooi K.-B. (2024). Can you resist the virtual temptations? Unveiling impulsive buying in Metaverse retail. Asia Pac. J. Mark. Logist. 36, 2259–2280. doi: 10.1108/APJML-09-2023-0911

[ref21] de Martínez García Leaniz P. Herrero Crespo Á. Gómez López R. (2018). Customer responses to environmentally certified hotels: the moderating effect of environmental consciousness on the formation of behavioral intentions. J. Sustain. Tour. 26, 1160–1177. doi: 10.1080/09669582.2017.1349775

[ref23] Deng Y. Shen H. Ji X. (2024). Exploring virtual fashion consumption through the emotional three-level theory: reflections on sustainable consumer behavior. Sustainability 16:5818. doi: 10.3390/su16135818

[ref24] Ding J. Lee E.-S. (2024). Promoting consumers’ sustainable consumption of online retail cold chain logistics services: extended applications of SOR and cognitive-affective-conative theories. Behav. Sci. 14:771. doi: 10.3390/bs14090771, 39335986 PMC11428570

[ref25] Fan X. Chai Z. Deng N. Dong X. (2020). Adoption of augmented reality in online retailing and consumers’ product attitude: a cognitive perspective. J. Retail. Consum. Serv. 53:101986. doi: 10.1016/j.jretconser.2019.101986

[ref26] Felzmann H. Fosch Villaronga E. Lutz C. Tamò-Larrieux A. (2019). Transparency you can trust: transparency requirements for artificial intelligence between legal norms and contextual concerns. Big Data Soc. 6:2053951719860542. doi: 10.1177/2053951719860542

[ref27] Fornell C. Larcker D. F. (1981). Evaluating structural equation models with unobservable variables and measurement error. J. Mark. Res. 18, 39–50. doi: 10.1177/002224378101800104

[ref28] Foroughi B. Naghmeh-Abbaspour B. Wen J. Ghobakhloo M. Al-Emran M. Al-Sharafi M. A. (2025). Determinants of generative AI in promoting green purchasing behavior: a hybrid partial least squares–artificial neural network approach. Bus. Strat. Environ. 34, 4072–4094. doi: 10.1002/bse.4186

[ref29] Gao Y. Liang J. (2025). The impact of AI-powered try-on technology on online consumers’ impulsive buying intention: the moderating role of brand trust. Sustainability 17:7. doi: 10.3390/su17072789

[ref31] Geng R. Chen X. Wang S. (2023). Wear in or wear out: how consumers respond to repetitive influencer marketing. Internet Res. 34, 810–848. doi: 10.1108/INTR-01-2022-0075

[ref32] George A. S. (2024). The hidden cost: understanding the environmental impact of online purchase returns. Partners Univ. Innov. Res. Public. 2, 132–149. doi: 10.5281/zenodo.10674476

[ref33] Gulati S. McDonagh J. Sousa S. Lamas D. (2024). Trust models and theories in human–computer interaction: a systematic literature review. Comput. Hum. Behav. Rep. 16:100495. doi: 10.1016/j.chbr.2024.100495

[ref34] Ham M. Jeger M. Frajman Ivković A. (2015). The role of subjective norms in forming the intention to purchase green food. Econ. Res.-Ekon. Istraz. 28, 738–748. doi: 10.1080/1331677X.2015.1083875

[ref35] Hilken T. Keeling D. I. de Ruyter K. Mahr D. Chylinski M. (2020). Seeing eye to eye: social augmented reality and shared decision making in the marketplace. J. Acad. Mark. Sci. 48, 143–164. doi: 10.1007/s11747-019-00688-0

[ref36] Hipólito F. Dias Á. Pereira L. (2025). Influence of consumer trust, return policy, and risk perception on satisfaction with the online shopping experience. Systems 13:158. doi: 10.3390/systems13030158

[ref37] Ho J.-Y. Ju G. Hong S. An J. Lee C. C. (2025). Factors influencing customer satisfaction with AR shopping assistant applications in E-commerce: an empirical analysis utilizing text-mining techniques. Aslib J. Inf. Manag. 77, 239–259. doi: 10.1108/AJIM-03-2023-0089

[ref38] Hu Y. Xiao Y. Hua Y. Fan Y. Li F. (2024). The more realism, the better? How does the realism of AI customer service agents influence customer satisfaction and repeat purchase intention in service recovery. Behav. Sci. 14:1182. doi: 10.3390/bs14121182, 39767323 PMC11673245

[ref39] Ivanov A. Head M. Biela C. (2022). Mobile shopping decision comfort using augmented reality: the effects of perceived augmentation and haptic imagery. Asia Pac. J. Mark. Logist. 35, 1917–1934. doi: 10.1108/APJML-06-2022-0518

[ref40] Jung J. H. Choi Y. J. Oh K. W. (2020). Influencing factors of Chinese consumers’ purchase intention to sustainable apparel products: exploring consumer ‘attitude–behavioral intention’ gap. Sustainability 12:1770. doi: 10.3390/su12051770

[ref41] Kang H. J. Shin J.-h. Ponto K. (2020). How 3D virtual reality stores can shape consumer purchase decisions: the roles of informativeness and playfulness. J. Interact. Mark. 49, 70–85. doi: 10.1016/j.intmar.2019.07.002

[ref42] Kim I. Ki C.-W. Lee H. Kim Y.-K. (2024a). Virtual influencer marketing: evaluating the influence of virtual influencers’ form realism and behavioral realism on consumer ambivalence and marketing performance. J. Bus. Res. 176:114611. doi: 10.1016/j.jbusres.2024.114611

[ref43] Kim S. Park H. Lim R. E. (2024b). Unveiling product imagination and decision comfort through personalized virtual try-on: the moderating role of spatial processing perception. J. Res. Interact. Mark. 19, 387–407. doi: 10.1108/JRIM-01-2024-0015

[ref44] Koay K. Y. Tey J. (2025). Understanding the influence of physical servicescape on consumers’ intentions to revisit dessert stores using the SOR model. J. Int. Food Agribus. Mark. 37, 467–488. doi: 10.1080/08974438.2024.2362189

[ref45] Kokash H. A. Ahsan M. N. Hafeez M. H. Khattak A. N. Ahmed S. Alam S. S. (2025). Environmental concern and digital engagement as drivers of circular product buying intention in Malaysia: an integrated S-O-R, TPB, and DOI perspective. Circ. Econ. Sustain. 5, 5429–5454. doi: 10.1007/s43615-025-00693-2

[ref46] Kolańska-Stronka M. Singh B. (2024). Five decades of self-congruity in consumer behaviour research: a systematic review and future research agenda. Int. J. Consum. Stud. 48:e12983. doi: 10.1111/ijcs.12983

[ref47] Kumar L. Hajari V. R. (2024). “AI algorithms for personalization: recommender systems, predictive analytics, and beyond.” SSRN Scholarly Paper No. 5051557. Social Science Research Network. Available online at: https://papers.ssrn.com/abstract=5051557 (Accessed October 11, 2025).

[ref48] Lau O. Ki C.-W. (2021). Can consumers’ gamified, personalized, and engaging experiences with VR fashion apps increase in-app purchase intention by fulfilling needs? Fash. Text. 8:36. doi: 10.1186/s40691-021-00270-9

[ref49] Lazaric N. Le Guel F. Belin J. Oltra V. Lavaud S. Douai A. (2020). Determinants of sustainable consumption in France: the importance of social influence and environmental values. J. Evol. Econ. 30, 1337–1366. doi: 10.1007/s00191-019-00654-7

[ref51] Li Y. Shan B. (2025). Exploring the role of health consciousness and environmental awareness in purchase intentions for green-packaged organic foods: An extended TPB model. Front. Nutr. 12:1528016. doi: 10.3389/fnut.2025.1528016, 40110167 PMC11921780

[ref52] Li S. Ye R. Dou C. Li J. Yang J. (2026). Constraints on youth participation in evening schools: empirical evidence from Shenyang, China. Sustainability 18:413. doi: 10.3390/su18010413

[ref53] Li Y. Zhang C. Shen T. Chen X. (2025). Seeing is believing: the impact of AI magic mirror on consumer purchase intentions in medical aesthetic services. J. Theor. Appl. Electron. Commer. Res. 20:205. doi: 10.3390/jtaer20030205

[ref54] Li X. Zhao X. Xu W. Pu W. (2020). Measuring ease of use of mobile applications in E-commerce retailing from the perspective of consumer online shopping behaviour patterns. J. Retail. Consum. Serv. 55:102093. doi: 10.1016/j.jretconser.2020.102093

[ref55] Liao C.-H. (2024). Exploring social media determinants in fostering pro-environmental behavior: insights from social impact theory and the theory of planned behavior. Front. Psychol. 15:1445549. doi: 10.3389/fpsyg.2024.1445549, 39144588 PMC11322476

[ref56] Lin S.-T. Niu H.-J. (2018). Green consumption: environmental knowledge, environmental consciousness, social norms, and purchasing behavior. Bus. Strateg. Environ. 27, 1679–1688. doi: 10.1002/bse.2233

[ref57] Liu H. Zhang P. Cheng H. Hasan N. Chiong R. (2025). Impact of AI-generated virtual streamer interaction on consumer purchase intention: a focus on social presence and perceived value. J. Retail. Consum. Serv. 85:104290. doi: 10.1016/j.jretconser.2025.104290

[ref22] Matos Celso Augusto de Luppi Lierge Veiga Ricardo Teixeira (2025) Assessing the intention-behavior gap in the pro-environmental behavior context: a longitudinal study about water conservation J. Clean. Prod. 524:146499 doi: 10.1016/j.jclepro.2025.146499

[ref58] McLean G. Wilson A. (2019). Shopping in the digital world: examining customer engagement through augmented reality mobile applications. Comput. Hum. Behav. 101, 210–224. doi: 10.1016/j.chb.2019.07.002

[ref59] Mollel J. Chen Y. (2025). Does AR virtual try-on boost online apparel purchase intentions? An integration of TAM, IDT, FIT perception and body esteem. Young Consum. 26, 385–402. doi: 10.1108/YC-08-2024-2196

[ref60] Mouloudj K. Aprile M. C. Bouarar A. C. Njoku A. Evans M. A. Oanh L. V. L. . (2025). Investigating antecedents of intention to use green Agri-food delivery apps: merging TPB with trust and electronic word of mouth. Sustainability 17:3717. doi: 10.3390/su17083717

[ref61] Mustafy T. Rahman M. T. U. (2024). “Spss,” in Statistics and Data Analysis for Engineers and Scientists, eds. Mustafy T. Rahman M. T. U. (Singapore: Springer Nature).

[ref62] Nguyễn T. N. Phan M. D. T. Nguyet P. T. A. (2025). The impact of environmental factors on green purchase intention and green purchase behavior: the moderating role of Price sensitivity. Management 2025, 597–624. doi: 10.58691/man/205146

[ref63] Oktavia P. I. Zulbainarni N. Hartono S. (2025). Generation z’s impulsive buying behavior on Shopee live: a stimulus-organism-response model. J. Bus. Manage. Rev. 6, 1055–1072. doi: 10.47153/jbmr.v6i8.1673

[ref64] Pantano E. Pizzi G. (2020). Forecasting artificial intelligence on online customer assistance: evidence from Chatbot patents analysis. J. Retail. Consum. Serv. 55:102096. doi: 10.1016/j.jretconser.2020.102096

[ref65] Patil D. (2024). Artificial intelligence in retail and E-commerce: enhancing customer experience through personalization, predictive analytics, and real-time engagement. Soc. Sci. Res. Netw. doi: 10.2139/ssrn.5057420

[ref66] Perumallaplli R. (2012). AI-enhanced personalization in E-commerce: redefining customer interaction. Soc. Sci. Res. Netw. doi: 10.2139/ssrn.5228719

[ref67] Phan T. A. Bui V. D. (2025). AI with a heart: how perceived authenticity and warmth shape Trust in Healthcare Chatbots. J. Mark. Commun., 1–21. doi: 10.1080/13527266.2025.2508887

[ref68] Phan T. A. Nguyen T. H.-T. Nguyen C. (2025). ‘Hey AI, what should I eat?’ Navigating skepticism and trust in AI-powered meal recommendations through personalized persuasion. Int. J. Hum. Comput. Interact., 1–13. doi: 10.1080/10447318.2025.2575098

[ref69] Phan T. A. Phuoc T. H. Trang N. T. (2026). AI, trust, and risk: decoding what really drives financial adoptionAI. Strategic Bus. Res. 2:100031. doi: 10.1016/j.sbr.2025.100031

[ref70] Pillarisetty R. Mishra P. (2021). Understanding the effect of AI and enabled technologies on customer experience in online fashion retail: An empirical study. J. Manag. 15, 1–14.

[ref71] Rajagopal Rajagopal A. (2023). ‘Seeing is experiencing’: impact of showcasing fashion merchandise on digital platforms. Qual. Mark. Res. Int. J. 26, 214–231. doi: 10.1108/QMR-05-2022-0080

[ref72] Sapp S. G. Harrod W. J. Zhao L. (1994). Socially constructed subjective norms and subjective norm-behavior consistency. Soc. Behav. Pers. 22, 31–40. doi: 10.2224/sbp.1994.22.1.31

[ref73] Sarkis N. Jabbour Al Maalouf N. Saliba E. Azizi J. (2025). The impact of augmented reality within the fashion industry on purchase decisions, customer engagement, and brand loyalty. Int. J. Fashion Des. Technol. Educ. 1–10. doi: 10.1080/17543266.2025.2470187

[ref74] Sharma N. Paço A. (2025). AI and emotions: enhancing green intentions through personalized recommendations—a mediated moderation analysis. AI & Soc. 40, 2821–2838. doi: 10.1007/s00146-024-02031-w

[ref75] Shen M. Wang J. (2022). The impact of pro-environmental awareness components on green consumption behavior: the moderation effect of consumer perceived cost, policy incentives, and face culture. Front. Psychol. 13:580823. doi: 10.3389/fpsyg.2022.580823, 35795420 PMC9252608

[ref76] Singh B. Chandra S. (2026). “AI-powered decision-making in self-service technologies: ethical and transparent AI in customer interactions,” in Navigating the Self-Service Revolution With Smart Machines, (Hershey, PA, USA: IGI Global Scientific Publishing).

[ref77] Sohaib O. Alshemeili A. Bhatti T.. (2025). Exploring AI-enabled green marketing and green intention: An integrated PLS-SEM and NCA approach. Cleaner and Responsible Consumption, 17:100269. doi: 10.1016/j.clrc.2025.100269

[ref78] Spais G. Jain V. (2025). Consumer behavior’s evolution, emergence, and future in the AI age through the lens of MR, VR, XR, metaverse, and robotics. J. Consum. Behav. 24, 1275–1299. doi: 10.1002/cb.2468

[ref79] Sujood N. B. Siddiqui S. (2022). Consumers’ intention towards the use of smart technologies in tourism and hospitality (T&H) industry: a deeper insight into the integration of TAM, TPB and trust. J. Hosp. Tour. Insights 7, 1412–1434. doi: 10.1108/JHTI-06-2022-0267

[ref80] Sun Y. Xing J. (2022). The impact of social media information sharing on the green purchase intention among generation Z. Sustainability 14:6879. doi: 10.3390/su14116879

[ref81] Sundar S. S. (2020). Rise of machine agency: a framework for studying the psychology of human–AI interaction (HAII). J. Comput.-Mediat. Commun. 25, 74–88. doi: 10.1093/jcmc/zmz026

[ref82] Surbakti F. P. S. Hutahaean H. A. Inderawati M. M. W. Madjid J. M. Sely L. E. Yee Y.-M. (2025). How AI-driven personalization shapes green purchasing behavior among youth in Java Island. Sustainability 17:9600. doi: 10.3390/su17219600

[ref83] Suresh A. Soni G. (2025). “Consumer-centric innovation in smart retail AR, VR, and industry 4.0: post-COVID-19 perspectives,” in Retail Innovations in Business Models, (Hershey, PA, USA: IGI Global Scientific Publishing).

[ref84] Sweller J. (2011). “CHAPTER TWO - cognitive load theory,” in Psychology of Learning and Motivation, eds. Mestre J. P. Ross B. H., vol. 55 (Academic Press).

[ref85] Tandon U. (2023). Chatbots, virtual-try-on (VTO), e-WOM: modeling the determinants of attitude’ and continued intention with PEEIM as moderator in online shopping. Glob. Knowl. Mem. Commun. 74, 147–165. doi: 10.1108/GKMC-06-2022-0125

[ref86] Testa D. S. Bakhshian S. Eike R. (2021). Engaging consumers with sustainable fashion on Instagram. J. Fash. Mark. Manag. 25, 569–584. doi: 10.1108/JFMM-11-2019-0266

[ref87] Varshneya G. Pandey S. K. Das G. (2017). Impact of social influence and green consumption values on purchase intention of organic clothing: a study on collectivist developing economy. Glob. Bus. Rev. 18, 478–492. doi: 10.1177/0972150916668620

[ref88] Veltri G. A. Lupiáñez-Villanueva F. Folkvord F. Theben A. Gaskell G. (2023). The impact of online platform transparency of information on consumers’ choices. Behav. Public Policy 7, 55–82. doi: 10.1017/bpp.2020.11

[ref89] Vlačić B. Corbo L. Costa e Silva S. Dabić M. (2021). The evolving role of artificial intelligence in marketing: a review and research agenda. J. Bus. Res. 128, 187–203. doi: 10.1016/j.jbusres.2021.01.055

[ref90] Wallace E. Buil I. (2023). Antecedents and consequences of conspicuous green behavior on social media: incorporating the virtual self-identity into the theory of planned behavior. J. Bus. Res. 157:113549. doi: 10.1016/j.jbusres.2022.113549

[ref91] Wang C. Fu Y. Yin Z. (2026). Platform-led or seller-led? Optimal ex ante information delivery strategy for online retail channels with product match uncertainty. Int. Trans. Oper. Res. 33, 1232–1268. doi: 10.1111/itor.13548

[ref92] Wu Y. Lee Y.-S. (2025). A study on the impact of the consumption value of sustainable fashion products on purchase intention based on the theory of planned behavior. Sustainability 17:4278. doi: 10.3390/su17104278

[ref93] Xie S. Madni G. R. (2023). Impact of social media on young generation’s green consumption behavior through subjective norms and perceived green value. Sustainability 15:3739. doi: 10.3390/su15043739

[ref94] Yang H. Kim Y. (2024). Southeast Asian consumer acceptance of 3D virtual fitting technologies in cross-border online shopping. Fash. Text. 11:6. doi: 10.1186/s40691-024-00372-0

[ref95] Yin J. Qiu X. Wang Y. (2025). The impact of AI-personalized recommendations on clicking intentions: evidence from Chinese E-commerce. J. Theor. Appl. Electron. Commer. Res. 20:21. doi: 10.3390/jtaer20010021

[ref96] Yrjölä M. Rintamäki T. Saarijärvi H. Joensuu J. (2017). Consumer-to-consumer e-commerce: outcomes and implications. Int. Rev. Retail Distrib. Consum. Res. 27, 300–315. doi: 10.1080/09593969.2017.1314864

[ref97] Yue B. Sheng G. She S. Xu J. (2020). Impact of consumer environmental responsibility on green consumption behavior in China: the role of environmental concern and price sensitivity. Sustainability 12:2074. doi: 10.3390/su12052074

[ref98] Yusuf N. P. (2025). Influence of Ease of Use and Trust of Artificial Intelligence on Customer Retention: A Study of Dublin’s Hospitality Industry. Masters, Dublin: National College of Ireland.

[ref99] Zhang Y. Jiang S. (2025). Pro-environmental personal norms and subjective norms related to AI-driven green entrepreneurship intention: a qualitative insight to explore students in higher education institutions. Afr. Educ. Res. J. 13, 83–99.

[ref100] Zhang S. Li J. Shi L. Ding M. Nguyen D. C. Chen W. . (2025). Industrial metaverse: enabling technologies, open problems, and future trends. IEEE Commun Surv Tutor 28, 2771–2804. doi: 10.1109/COMST.2025.3563919

[ref101] Zhang W. Wang Y. (2023). What drives customers to use virtual fitting rooms? The moderating effect of fashion consciousness. J. Fash. Mark. Manag. 28, 929–949. doi: 10.1108/JFMM-04-2023-0099

[ref102] Zhang J. Wang X. Lu J. Liu L. Feng Y. (2024). The impact of emotional expression by artificial intelligence recommendation chatbots on perceived humanness and social interactivity. Decis. Support. Syst. 187:114347. doi: 10.1016/j.dss.2024.114347

[ref103] Zhao Y. Li Y. Wang N. Zhou R. Luo X. (2022). A meta-analysis of online impulsive buying and the moderating effect of economic development level. Inf. Syst. Front. 24, 1667–1688. doi: 10.1007/s10796-021-10170-4, 34393617 PMC8355873

[ref104] Zhu J. Jiang L. Dou W. Wang V. L. Zhou L. (2024). Beyond the ‘like’ button: ideal social self-congruity’s role in shaping consumer behaviors on social media. J. Res. Interact. Mark. 19, 969–983. doi: 10.1108/JRIM-04-2024-0190

[ref105] Zhuo Zihui Ren Zheng Zhu Zhiwen. (2023). “Attitude-behavior gap in green consumption behavior: a review.” SSRN Scholarly Paper No. 4673201. Social Science Research Network, December 22. Available online at: https://papers.ssrn.com/abstract=4673201 (Accessed December 22, 2023).

