# Supplementary material for: AI-based virtual try-on technology and the activation of green psychological mechanisms: evidence from a digital consumer environment
Source: Front Psychol. 2026 Mar 18;17:1750808. doi: 10.3389/fpsyg.2026.1750808 (PMC13038539; doi:10.3389/fpsyg.2026.1750808)
Supplement: Supplementary file 1 [file Table_2.docx]

# Appendix A. Measurement

| **Constructs** | **Item** | **Measurement Item** | **Source** | **Adaptation** |
| --- | --- | --- | --- | --- |
| Visual Realism (VR) | VR1 | I find the garments presented through VTO be highly vivid. | (McLean and Wilson 2019) | Reworded for VTO context |
|  | VR2 | The texture and color of the virtual garments closely resemble those of real clothing. | (Gao and Liang 2025) | Minor wording adaptation |
|  | VR3 | The virtual model’s body features and overall proportions closely resemble my own. | (Gao and Liang 2025) | Minor wording adaptation |
|  | VR4 | The virtual model’s body features and overall proportions closely resemble my own. | (Kang et al. 2020) | Reworded for VTO context |
| Personalized Recommendation (PR) | PR1 | The clothing recommended by the system aligns well with my style preferences. | (Yin et al. 2025) | Reworded for VTO context |
|  | PR 2 | The VTO system accurately identifies my body shape and size. | (Gao and Liang 2025) | Minor wording adaptation |
|  | PR 3 | The system’s recommendations feel “just right” for me. | (Gao and Liu 2022) | Reworded for VTO context |
|  | PR 4 | I feel that the system understands my personalized needs. | (Yin et al. 2025) | Minor wording adaptation |
| Self-congruity（SC） | SC 1 | The virtual avatar closely resembles my real-world appearance. | (Bowden et al. 2025) | Minor wording adaptation |
|  | SC 2 | I believe the virtual model accurately reflects my style and temperament. | (Mollel and Chen 2025) | Reworded for VTO context |
|  | SC 3 | The virtual image effectively expresses my self-identity. | (Bowden et al. 2025) | Reworded for VTO context |
|  | SC 4 | The VTO effect is consistent with my real-life dressing experience. | (Bowden et al. 2025) | Reworded for VTO context |
| Ease of use (EOU) | EOU 1 | The VTO system is very easy to operate and understand. | (Zhang and Wang 2023) | Reworded for VTO context |
|  | EOU 2 | I can easily learn how to use the VTO feature. | (McLean and Wilson 2019) | No conceptual change |
|  | EOU 3 | The entire try-on process is efficient and effortless. | (Gao and Liang 2025) | No conceptual change |
|  | EOU 4 | Using the VTO technology makes me feel convenient and pleasant. | (Zhao et al. 2022) | Reworded for VTO context |
| Feedback Transparency (FT) | FT 1 | The system clearly presents the basis for generating VTO results. | (Cheng 2026) | Reworded for VTO context |
|  | FT 2 | I can understand the reasons behind the system’s recommendations. | (Gao and Liang 2025) | Reworded for VTO context |
|  | FT3 | The feedback provided by the system increases my trust in the try-on results. | (Pillarisetty and Mishra, n.d.) | Reworded for VTO context |
|  | FT 4 | The system’s information presentation makes me feel that it is transparent and reliable. | (Kumar and Hajari 2024) | Reworded for VTO context |
| Social Interactivity (SI) | SI 1 | I can easily share the VTO results with my friends. | (Gao and Liang 2025) | Minor wording adaptation |
|  | SI 2 | The VTO experience makes me want to interact and communicate with others. | (McLean and Wilson 2019) | Reworded for VTO context |
|  | SI 3 | I enjoy showcasing my VTO experiences on social media. | (Tandon 2023) | Reworded for VTO context |
|  | SI 4 | Sharing my try-on experience with others increases my desire to purchase the clothing. | (Gao and Liang 2025) | Minor wording adaptation |
| Attitude（AT） | AT 1 | I believe that rational purchasing and waste reduction are positive behaviors. | (Tandon 2023) | Reworded for VTO context |
|  | AT 2 | I hold a favorable attitude toward reducing product returns through VTO. | (Angelbratt 2025) | Reworded for VTO context |
|  | AT 3 | Using VTO contributes to a more sustainable way of shopping. | (Angelbratt 2025) | Reworded for VTO context |
|  | AT 4 | I enjoy engaging in environmentally friendly consumption through technological means. | (Sohaib et al. 2025) | Reworded for VTO context |
| Perceived Behavioral Control（PBC） | PBC1 | I believe I am capable of making sustainable shopping decisions. | (Sharma and Paço 2025) | Reworded for VTO context |
|  | PBC2 | Using VTO makes me feel that green consumption is easier to achieve. | (Surbakti et al. 2025) | Reworded for VTO context |
|  | PBC 3 | I can easily determine whether the clothing fits me through VTO. | (Gao and Liang 2025) | Minor wording adaptation |
|  | PBC4 | I am confident in choosing products that are both fashionable and environmentally friendly. | (Wu and Lee 2025) | Reworded for VTO context |
| Subjective Norm (SN) | SN 1 | People around me believe that environmentally friendly consumption is commendable. | (Surbakti et al. 2025) | Reworded for VTO context |
|  | SN 2 | My friends and family encourage me to shop rationally and sustainably. | (Surbakti et al. 2025) | Reworded for VTO context |
|  | SN 3 | I care about others’ opinions regarding my green consumption behavior. | (Wu and Lee 2025) | Reworded for VTO context |
|  | SN 4 | When I use the VTO function, I hope others will approve of this behavior. | (Wu and Lee 2025) | Reworded for VTO context |
| Green Purchase Intention（GPI） | GPI 1 | I am willing to purchase products that help reduce waste in the future. | (Surbakti et al. 2025) | Minor wording adaptation |
|  | GPI 2 | I plan to prioritize green and sustainable brands or products when shopping. | (Surbakti et al. 2025) | Minor wording adaptation |
|  | GPI 3 | I actively seek information about a product’s environmental attributes or sustainable production to support my purchase decisions. | (Wu and Lee 2025) | Reworded for VTO context |
| Environmental Awareness（EA） | EA 1 | I am aware that my personal shopping behavior has a direct impact on the environment. | (Surbakti et al. 2025) | Minor wording adaptation |
|  | EA 2 | I believe consumers have a responsibility to reduce environmental pollution through their purchase decisions. | (Wu and Lee 2025) | Reworded for VTO context |
|  | EA 3 | I pay attention to companies’ and brands’ actual practices regarding environmental protection and sustainability. | (Wu and Lee 2025) | Reworded for VTO context |
